# Knock‐down of gene expression throughout meiosis and pollen formation by virus‐induced gene silencing in *Arabidopsis thaliana*

**DOI:** 10.1111/tpj.15733

**Published:** 2022-06-18

**Authors:** Vanesa Calvo‐Baltanás, Joke De Jaeger‐Braet, Wei Yuan Cher, Nils Schönbeck, Eunyoung Chae, Arp Schnittger, Erik Wijnker

**Affiliations:** ^1^ Laboratory of Genetics Wageningen University & Research Droevendaalsesteeg 1 Wageningen 6700 AA the Netherlands; ^2^ Department of Developmental Biology, Institut für Pflanzenwissenschaften und Mikrobiologie University of Hamburg Ohnhorststrasse 18 Hamburg 22609 Germany; ^3^ Department of Biological Sciences National University of Singapore 14 Science Drive 4 Singapore 117543 Singapore; ^4^ A*STAR, Institute of Molecular and Cell Biology (IMCB) 61 Biopolis Drive Proteos 138673 Singapore; ^5^ UKE Martinistrasse 52 20251 Hamburg Germany

**Keywords:** virus‐induced gene silencing, increase of crossover recombination, unreduced gametes, pollen tetrads, *Arabidopsis thaliana*, meiosis

## Abstract

Through the inactivation of genes that act during meiosis it is possible to direct the genetic make‐up of plants in subsequent generations and optimize breeding schemes. Offspring may show higher recombination of parental alleles resulting from elevated crossover (CO) incidence, or by omission of meiotic divisions, offspring may become polyploid. However, stable mutations in genes essential for recombination, or for either one of the two meiotic divisions, can have pleiotropic effects on plant morphology and line stability, for instance by causing lower fertility. Therefore, it is often favorable to temporarily change gene expression during meiosis rather than relying on stable null mutants. It was previously shown that virus‐induced gene silencing (VIGS) can be used to transiently reduce CO frequencies. We asked if VIGS could also be used to modify other processes throughout meiosis and during pollen formation in *Arabidopsis thaliana*. Here, we show that VIGS‐mediated knock‐down of *FIGL1*, *RECQ4A/B*, *OSD1* and *QRT2* can induce (i) an increase in chiasma numbers, (ii) unreduced gametes and (iii) pollen tetrads. We further show that VIGS can target both sexes and different genetic backgrounds and can simultaneously silence different gene copies. The successful knock‐down of these genes in *A. thaliana* suggests that VIGS can be exploited to manipulate any process during or shortly after meiosis. Hence, the transient induction of changes in inheritance patterns can be used as a powerful tool for applied research and biotechnological applications.

## Significance Statement

The present work demonstrates that VIGS is an efficient tool to quickly accomplish breeding aims such as an increase in crossover recombination, the generation of polyploid offspring and modification of pollen‐specific processes.

## INTRODUCTION

Research over the last years has highlighted how the targeted modification of plant meiosis can be instrumental to develop new breeding strategies and facilitate the fast production of mapping populations (Lambing et al., 2017; Wijnker & de Jong, 2008). For instance, the increase of meiotic recombination frequencies can be used to expedite fine mapping of traits through the generation of mapping populations with high crossover (CO) numbers. Conversely, the decrease of CO frequencies helps the development of chromosome substitution lines (CSLs) that can be employed to rapidly trace the rough location of loci of interest in the genome (Wijnen et al., 2018). In other applications, the formation of diploid, i.e., unreduced gametes can be used to produce polyploid offspring (Brownfield & Köhler, 2011; Cromer et al., 2012).

In practice, however, changing meiosis for breeding applications is a challenge since meiotic mutants are often sterile or display other pleiotropic defects, such as compromised DNA damage repair (Da Ines et al., 2013; Knoll et al., 2014; Kwon et al., 2013; Lu et al., 2008; Luo et al., 2013; Xu et al., 2018; Zhang, Zhang, et al., 2017). Moreover, developing mapping populations in mutant backgrounds is often impractical, especially when these mutations affect seed set or plant vigor. To overcome these negative effects, a transient gene silencing approach can be applied to exploit the full potential of modulating meiosis for breeding purposes. The feasibility of such approaches has been demonstrated by the dominant knock‐down of genes expressed in meiosis using stably transformed RNA interference (RNAi) constructs (Boden et al., 2009; Siaud et al., 2004; Wijnker et al., 2012), or through the transient silencing of genes involved in meiotic recombination by using virus‐induced gene silencing (VIGS) (Bennypaul et al., 2012; Bhullar et al., 2014; Calvo‐Baltanás et al., 2020; Raz et al., 2021). An added benefit of the use of transient silencing is that it is possible to obtain transgene‐free offspring. RNAi constructs act dominantly, and will segregate in offspring, whereas VIGS can be applied such that no stable integration into the plant genomes is required (Calvo‐Baltanás et al., 2020; Senthil‐Kumar & Mysore, 2011; Wijnker et al., 2012). For these reasons transient silencing may be preferred over stable transformation in breeding schemes.

Both techniques, RNAi and VIGS, lead to post‐transcriptional silencing of endogenous genes by exploiting the RNAi pathway. In both cases the silencing relies on the generation of double‐stranded RNA specimens (dsRNAs) homologous to the mRNA of a target gene. The presence of dsRNAs in the host cells triggers an immune response leading to cleavage of these dsRNAs into 21–24‐nucleotide‐long, single‐stranded RNA molecules known as small interfering RNAs (siRNAs) (Becker & Lange, 2010; Padmanabhan & Dinesh‐Kumar, 2009; Zhang et al., 2019). The generated siRNAs are loaded onto the RISC complex which recognizes and cleaves sequences complementary to these siRNAs. Cleavage of complementary RNA strands (i.e., not only viral RNAs but also endogenous plant mRNAs) induces the knock‐down of the target gene (Mermigka et al., 2016). Whereas RNAi techniques require the stable transformation of plant reproductive tissues, VIGS depends on the transient expression of an active, autonomous virus in the host plant (Ruiz et al., 1998). Hence, VIGS circumvents the often lengthy and laborious generation of stable transgenic plants.

The most popular VIGS system used in *Arabidopsis thaliana* is based on the Tobacco rattle virus (TRV), which consists of a positive‐sense, single‐stranded RNA viral genome (Ratcliff et al., 2001). For its application, the TRV viral genome is encoded by two DNA plasmids, TRV1 and TRV2, which are delivered through *Agrobacterium*‐mediated transformation of vegetative parts, typically leaves, of the host plant (Burch‐Smith et al., 2006; Ratcliff et al., 2001). TRV1 encodes genes for viral replication and movement, whereas TRV2 contains the genetic information for capsule proteins and other non‐structural proteins (Ratcliff et al., 2001). Short exogenous plant DNA sequences of target genes can be inserted into TRV2 as described in various publications (Burch‐Smith et al., 2004, 2006; Liu, Schiff, Marathe, 2002; Ratcliff et al., 2001; Senthil‐Kumar & Mysore, 2014). After *Agrobacterium*‐mediated plant transformation, an active virus is assembled and spreads through the plant, producing dsRNAs during viral reproduction within the host cells.

### The targeted modification of meiosis can optimize breeding schemes

Increasing CO recombination frequencies in heterozygous plants could facilitate the introgression of traits (by breaking up tight linkages) or the fine mapping of traits in offspring populations. Obvious targets for VIGS‐mediated increase of meiotic recombination frequencies are the suppressors of the interference‐independent class II CO pathway such as *FIGL1* (*FIDGETIN‐Like‐1*) and the RECQ helicases RECQ4A/RECQ4B. The *figl1* and *recq4a/recq4b* mutants in *A. thaliana* display a 1.8‐fold CO increase in Col/Landsberg *erecta* (L*er*) F_1_ and an up to sixfold increase in Col‐0, respectively (Girard et al., 2015; Séguéla‐Arnaud et al., 2015). These anti‐COs factors were initially identified through suppressor‐screens in which mutants of *FIGL1* and *RECQ4* rescued the semi‐sterile phenotype of *zmm* mutants, in which the class I CO pathway is defective (Girard et al., 2015; Séguéla‐Arnaud et al., 2015). The *zmm* mutants in *A. thaliana* typically show about 80% reduction in CO numbers, which leads to unbalanced chromosome segregation, high frequencies of non‐viable, aneuploid gametes and a reduced seed set (Chelysheva et al., 2012; Higgins et al., 2004, 2008; Lu et al., 2008; Mercier et al., 2005).

Another interesting aim in applied and fundamental research is the production of polyploid offspring. One possibility to do that is through the generation of unreduced gametes, which can be formed in plants when genes required during the first or the second meiotic division are mutated ( Brownfield & Köhler, 2011; D'Erfurth et al., 2008; De Storme & Geelen, 2011). For instance, OSD1 (OMISSION OF SECOND DIVISION 1), also known as GIGAS CELL 1 (GIG1), mediates meiotic progression by inhibiting APC/C (ANAPHASE PROMOTING COMPLEX/C), a key cell cycle regulator (Cromer et al., 2012; Iwata et al., 2011). Mutants of *OSD1* produce viable, balanced diploid gametes through a second meiotic division restitution, thus often referred to as second meiotic restitution (SDR) gametes (D'Erfurth et al., 2009). This mutation has, for example, been utilized in strategies to achieve clonal reproduction through seeds in *A. thaliana* and rice (*Oryza sativa*) (D'Erfurth et al., 2009; Marimuthu et al., 2011; Mieulet et al., 2016; Wang et al., 2019).

There are other genes that are not strictly required for meiosis, but that are instrumental to the study of meiotic segregation, such as the *QRT (QUARTET)* genes (Copenhaver et al., 2000; Francis et al., 2007). In *qrt* mutants, pollen tetrads are formed when the four viable and fertile male spores from a single meiosis are released as a single unit (Ogawa et al., 2013; Preuss et al., 1994). Pollen tetrad formation in *A. thaliana* occurs in the absence of any of the three *QRT* genes: *QRT1*, *QRT2* or *QRT3* (Francis et al., 2006; Rhee & Somerville, 1998; Rhee et al., 2003). Pollen tetrads have been helpful to assess the precise placement and frequencies of CO and gene conversions in *A. thaliana* and maize (*Zea mays*) (Li et al., 2015; Lu et al., 2012; Sun et al., 2012; Wijnker et al., 2013), to study CO recombination rates in wild‐type plants and various mutant backgrounds (Berchowitz & Copenhaver, 2007; Fernandes et al., 2017; Francis et al., 2007; Girard et al., 2015; Lim et al., 2020) and to identify factors involved in pollen development in *A. thaliana* (Roy & Copenhaver, 2011).

In this study, we evaluate the potential of VIGS to modify different aspects of meiosis and pollen formation in *A. thaliana*, which can be indicative of its feasibility to modulate meiosis in other species. We demonstrate that the TRV‐VIGS system can efficiently knock down essential regulators of meiotic recombination (*FIGL1* and *RECQ4A/B*) and genes acting during the second meiotic division (*OSD1*) and during early pollen development (*QRT2*). The silencing of these genes leads in each case to a phenocopy of their described mutant phenotypes: (i) an up to sixfold increase in chiasma numbers in the absence of *RECQ4* and *FIGL1*, (ii) the generation of unreduced gametes and polyploid offspring following the knock‐down of *OSD1* and (iii) pollen tetrad formation through the silencing of *QRT2*. Importantly, by silencing of *OSD1* we also illustrate that both male and female meiosis can be targeted using VIGS in different accessions of *A. thaliana*. Lastly, TRV‐mediated silencing of *QRT2* not only represents a useful tool to assist in recombination studies, but it also serves as a phenotypic marker to determine VIGS silencing efficiency in gametes over time.

## RESULTS

### Increase of meiotic recombination through VIGS‐mediated silencing of *FIGL1* and *RECQ4A/B*

We set out to explore if VIGS could be used to increase meiotic CO frequencies through the knock‐down of *FIGL1* and/or *RECQ4*. The VIGS‐mediated downregulation of gene expression is partial, and therefore successful silencing is best assessed when phenotypes are clear and unequivocal (Calvo‐Baltanás et al., 2020). Because an increase in meiotic recombination frequencies is not easily assessed in wild‐type plants, we followed a similar testing strategy as described by Girard et al. (2015) and Séguéla‐Arnaud et al. (2015): rescue the semi‐sterile phenotype of a *zmm* mutant (*msh4* −/− [hereafter *msh4*]) by suppressing the action of *FIGL1* and *RECQ4* using VIGS. The successful downregulation of these genes would increase CO formation, restore balanced chromosome segregation and increase seed set in siliques of VIGS‐treated *msh4* plants.

To knock down the expression of the single‐copy gene *FIGL1*, a 169‐bp fragment spanning exon 1 of *FIGL1* was selected and introduced into TRV2 (TRV‐*FIGL1*). For *RECQ4* two gene copies exist in *A. thaliana*: *RECQ4A* and its paralog, *RECQ4B*. Although in some accessions, like L*er*, only *RECQ4A* is functional, the downregulation of *RECQ4* expression in Col‐0 requires the simultaneous targeting of both *RECQ4A* and *RECQ4B* (Séguéla‐Arnaud et al., 2015). To this end we generated the TRV‐*RECQ4* vector carrying a 240‐bp fragment of *RECQ4A* spanning exon 13 and exon 14, which share 88% sequence homology with *RECQ4B*.

We first asked whether these constructs could induce an active virus to spread in plants. Both TRV1 and TRV2 vectors need to be co‐delivered and co‐transformed into leaf cells to achieve successful expression and the formation of an active virus (Ratcliff et al., 2001). We therefore tested if transcripts of either vector could be detected in the flower buds of infiltrated plants. The incipient inflorescences of VIGS‐treated Col‐0 *msh4* plants were harvested 4 weeks post‐infiltration and were used to perform semi‐quantitative RT‐PCR (sqRT‐PCR). As a positive control, we used a Col‐0 *msh4* plant infiltrated with TRV2 carrying an inactive fragment of the reporter gene encoding GUS (hereafter TRV‐*GUS*) (Tameling & Baulcombe, 2007). Two non‐infiltrated *msh4* plants served as negative controls (Figure S1a). The expression of TRV1 could be detected in all treated plants, as well as in the *msh4*::TRV‐*GUS* control, but not in the non‐infiltrated *msh4* mutants (Figure S1a). The expression of TRV2‐derived transcripts was detected in all treated plants, except for one plant (Plant #1) infiltrated with TRV‐*FIGL1*, indicating that target transcripts derived from TRV2 were not present in that inflorescence at the moment of sampling (Figure S1a).

In a second control experiment, we tested whether TRV‐*FIGL1* and/or TRV‐*RECQ4* would affect fertility of wild‐type plants. Because these constructs are intended to be used for the rescue of a semi‐sterile mutant phenotype of *msh4*, the constructs should not induce sterility through pleiotropic effects. This is not trivial, as mutations in *FIGL1* and *RECQ4* were reported to cause pollen abortion in Col‐L*er* hybrids and Col‐0 *figl1* mutants display a reduction of the seed set as compared to wild‐type controls (Fernandes et al., 2017). To this end, we infiltrated six Col‐0 wild‐type plants with TRV‐*FIGL1*, six others with TRV‐*RECQ4* and four plants with TRV‐*GUS*. A total of six non‐infiltrated Col‐0 wild‐type plants were used as controls (Data S1). Using a generalized linear mixed model (GLMM; see ‘Experimental Procedures’ section) we found no significant differences in the number of well‐developed seeds in siliques of plants infiltrated with TRV‐*FIGL1* (*n* = 60), TRV‐*RECQ4* (*n* = 60) or TRV‐*GUS* (*n* = 40) and non‐infiltrated controls (*n* = 60) (Figures S2 and S3, Table S1).

### The TRV‐VIGS constructs TRV‐*FIGL1* and TRV‐*RECQ4* lead to increased seed sets in *msh4* plants

**Figure 1:**
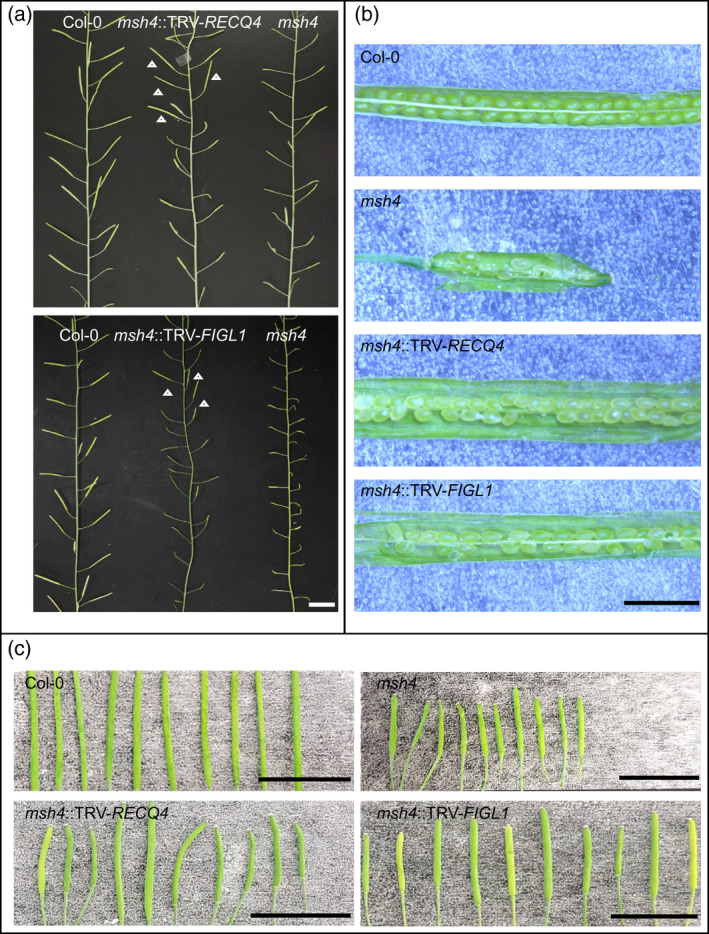
Inoculation of Col‐0 *msh4* plants with TRV‐*FIGL1* and TRV‐*RECQ4* results in partial rescue of the semi‐sterile phenotype. (a) Upper picture: Comparison between a wild‐type plant (left), a *msh4* mutant inoculated with TRV‐*RECQ4* (middle) and a *msh4* mutant (right). Inoculation of *msh4* with TRV‐*RECQ4* results in several long siliques (indicated by white arrowheads). Lower picture: The same comparison as in the upper picture (wild type left; treatment middle and *msh4* right) but now for the rescue of *msh4* using TRV‐*FIGL1*. Unusually long siliques in *msh4*::TRV‐*FIGL1* are indicated by white arrows. Scale bar, 1 cm. (b) Siliques selected from a single plant treated with TRV‐*FIGL1* and with TRV‐*RECQ4* show a clear rescue of the semi‐sterile phenotype in comparison with *msh4*, resembling the viable seed set found in Col‐0 controls. Scale bar, 0.2 cm. (c) A close‐up comparison of the 10 longest selected siliques from the same plant of a wild‐type control, a *msh4* control, a *msh4*::TRV‐*RECQ4* plant and a *msh4*::TRV‐*FIGL1* plant. While all the siliques from the wild‐type plant are long and regular, those selected from *msh4* are characteristically short due to the low number of seeds they contain. Siliques selected from *msh4*::TRV‐*RECQ4* and *msh4*::TRV‐*FIGL1* show that both long (increased seed set) and short siliques can be observed after silencing of either *FIGL1* or *RECQ4*. Scale bar, 1 cm. [Colour figure can be viewed at wileyonlinelibrary.com]

We asked if TRV‐*FIGL1* and TRV‐*RECQ4* could successfully downregulate *FIGL1* and *RECQ4A/B* by infiltrating Col‐0 *msh4* mutant plants. Effective silencing by either construct would elevate class II CO during meiosis (Girard et al., 2015; Séguéla‐Arnaud et al., 2015) and increase seed set in treated plants. Ten *msh4* plants were infiltrated with TRV‐*FIGL1* and 10 more with TRV‐*RECQ4*. In the same experiment we grew 13 non‐infiltrated *msh4* plants, four *msh4*::TRV‐*GUS* plants and four wild‐type Col‐0 plants as controls. Plants were grown under the same conditions and left to set seed. Where *msh4* mutant plants show the consistent formation of short siliques, a number of elongated siliques formed on the main inflorescence stem as well as on side branches of TRV‐*FIGL1*‐ and TRV‐*RECQ4*‐treated plants, which presumably contain a larger number of seeds in comparison to those produced by controls (Figure 1).

**Figure 2:**
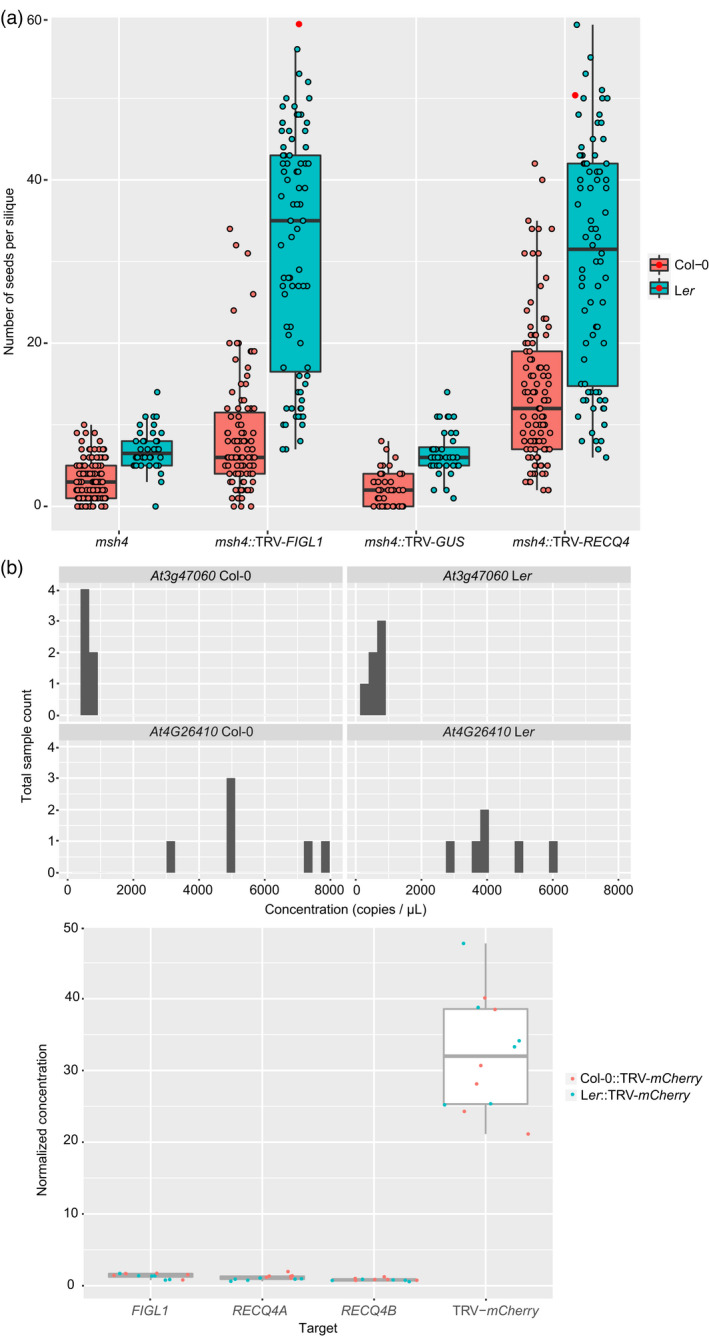
Silencing of *FIGL* or *RECQ4* rescues the semi‐sterile phenotype of *msh4* mutant plants in different genetic backgrounds. (a) The number of seeds per silique in *msh4*, *msh4*::TRV‐*FIGL1*, *msh4*::TRV‐*GUS* and *msh4*::TRV‐*RECQ4*, in the genetic backgrounds of Col‐0 (red) and L*er* (blue). For each plant, the seeds in the 10 longest siliques of the main stem were counted. Each individual silique is represented with a dot. Significant differences between TRV‐*FIGL1*‐ and TRV‐*RECQ4*‐inoculated plants and controls are observed for Col‐0 and L*er* (*P* < 0.001; Table S2). Note that the outliers are indicated with a red dot. Since our statistical analysis methods are sensitive to outliers, these data points are excluded from further data analysis as per the protocol described in Zuur et al. (2010). (b) Top panel: Exploratory data analysis to assess *At4G26410* and *AT3G47060* transcript stability in Col‐0::TRV‐*mCherry* and L*er*::TRV‐*mCherry*. *AT3G47060* presents lower variation across samples and is therefore used as a reference gene and for normalization. Bottom panel: Normalized expression level of TRV‐*mCherry* and endogenous *FIGL1*, *RECQ4A* and *RECQ4B* in inflorescences of Col‐0 (red) and L*er* (blue) plants infiltrated with TRV‐*mCherry*. No significant differences in TRV‐*mCherry* expression were detected between Col‐0 and L*er* samples (*P* > 0.05). Note that the TRV‐*mCherry* expression level is about 32 times higher than the expression level of endogenous genes. [Colour figure can be viewed at wileyonlinelibrary.com]

To confirm that elongated siliques observed in these plants corresponded to an increment in the number of seeds, we selected the 10 longest siliques that we could identify on the main inflorescence stem in all plants (*msh4*::TRV‐*FIGL1* [*n* = 100], *msh4*::TRV‐*RECQ4* [*n* = 100], *msh4*::TRV‐*GUS* [*n* = 40], *msh4* [*n* = 130]) and counted the number of seeds in each silique (Figures 1 and 2a, Figure S4, Data S1). In *msh4* plants, the mean number of seeds in the 10 longest siliques was 3.07 (SD = 2.39). In *msh4* plants infiltrated with TRV‐*GUS*, the average seed set was 2.38 seeds per silique (SD = 2.14), which is not significantly different (GLMM, *P* = 0.289; Table S2). Infiltration of *msh4* plants with either TRV‐*FIGL1* or TRV‐*RECQ4* led to significantly higher average seed set numbers (9.02 [SD = 8.62] and 14.3 [SD = 9.87] seeds per silique, respectively; GLMM, *P* < 0.001; Table S2). In addition, we found that in *msh4* controls, the maximum number of seeds per silique never exceeded 10 in any plant, whereas for instance, one plant of *msh4*::TRV‐*RECQ4* showed siliques with maximums of 52, 42 and 40 well‐developed seeds, and a single *msh4* plant treated with TRV‐*FIGL1* produced siliques with 58, 34 and 32 seeds (Data S1; Figure 2a). We therefore conclude that the constructs TRV‐*FIGL1* and TRV‐*RECQ4* can significantly increase seed set numbers in Col‐0 *msh4* plants.

Further, we evaluated whether silencing of *RECQ4* or *FIGL1* could lead to an increase in seed set in a different accession, so we executed similar experiments using *msh4* L*er* plants. We infiltrated eight *msh4* L*er* plants with TRV‐*RECQ4*, eight with TRV‐*FIGL1* and four with TRV‐*GUS* and grew four non‐infiltrated *msh4* L*er* plants for reference. We counted the number of seeds per silique in 10 siliques per plant − with the exception of *msh4* non‐infiltrated control (*n* = 38) and *msh4*::TRV‐*FIGL1* (*n* = 79) (Data S1, Figure 2a, Figures S5 and S6) and found that the results for *L*er are comparable to those of Col‐0. Both constructs, TRV‐*FIGL1* (mean = 31.4; SD = 14.42) and TRV‐*RECQ4* (mean = 30.2; SD = 14.35), but not TRV‐*GUS* (mean = 6.63; SD = 2.70), significantly increase the number of seeds per silique as compared to *msh4* controls (mean = 6.95; SD = 2.58) (GLMM, *P* < 0.001; Table S2, Figure 2a). In conclusion, these results show that both TRV‐*RECQ4* and TRV‐*FIGL1* significantly increased the seed set in *msh4* mutants of Col‐0 and L*er*.

We noted that silencing of either *RECQ4* or *FIGL1* led to a significantly higher increase in seed set in L*er* than in Col‐0 (Figure 2a). The L*er msh4* mutant already has a significantly higher seed set than the Col‐0 *msh4* mutant, but those differences are exacerbated upon treatment with TRV‐*FIGL1* and TRV‐*RECQ4*. We therefore asked if TRV expression differences might have caused these variations. To this end we infiltrated 2‐week‐old Col‐0 and L*er* plants with TRV‐*FIGL1* (*n* = 6), TRV‐*RECQ4* (*n* = 6) or TRV expressing an inactive fragment of mCherry (TRV‐*mCherry*) as control. We harvested the incipient inflorescences of all plants 3 weeks post‐infiltration and performed sqRT‐PCR to detect the expression of TRV2. We found that among all treated plants, two plants (one L*er*::TRV‐*RECQ4* and one Col‐0::TRV‐*FIGL1*) were negative for the expression of TRV2. We observed that TRV2 was similarly expressed in inflorescences of plants infiltrated with TRV‐*FIGL1*, TRV‐*RECQ4* and TRV‐*mCherry* (Figure S1b).

Because sqRT‐PCR only offers a semi‐quantitative approach to the expression of target genes, we decided to use a more accurate method to pinpoint possible differences in TRV expression between Col‐0 and L*er*. We carried out digital PCR (dPCR) assays to assess TRV viral load on the inflorescences obtained from L*er*::TRV‐*mCherry* (*n* = 6) and Col‐0::TRV‐*mCherry* (*n* = 6). For comparison and for normalization of the TRV2‐*mCherry* expression per sample, we used two reference genes: *At4G26410* and *At3G47060* (see ‘Experimental Procedures’ section). The absolute transcript abundance per sample and target can be found in Data S1 as the number of copies per microliter. To assess which of the two reference genes showed less variation in their expression levels, we performed exploratory data analysis, and found that *AT3G47060* transcript abundance was more stable across samples (Figure 2, Data S1). We then divided the absolute TRV2‐*mCherr*y concentration of each sample by the corresponding *AT3G47060* concentration and compared the normalized TRV2‐*mCherry* abundance between Col‐0 and L*er* (Figure 2b). The data were analyzed using the Mann–Whitney *U* test/Wilcoxon rank‐sum test, and we found no significant differences in TRV2 expression in inflorescences between Col‐0 and L*er* plants infiltrated with TRV‐*mCherry* (*W* = 13, *P* = 0.4848). These results show that high seed sets produced by L*er*::TRV‐*FIGL1* and L*er*::TRV‐*RECQ4* do not arise from differences in TRV expression between L*er* and Col‐0.

In our experiments, we observed that *msh4* plants infiltrated with TRV‐*FIGL1* and TRV‐*RECQ4* showed high variation regarding the silencing phenotype in both Col‐0 and L*er*; while some siliques of treated *msh4* plants showed seed sets as high as those produced by the wild type (Data S1), others did not present any signs of phenotypic rescue. These differences might be explained by too low TRV expression to affect all the cells in the inflorescence, causing incomplete silencing of the target gene. To test this hypothesis, we checked by dPCR if TRV‐*mCherry* expression was lower or higher than the endogenous expression of *FIGL1*, *RECQ4A* and *RECQ4B* using the samples obtained from Col‐0 and L*er* plants infiltrated with TRV‐*mCherry*. For this comparison, we divided the transcript abundance of each target gene by the corresponding *AT3G47060* abundance in each sample (Data S1). Surprisingly, we found that TRV2 expression was about 32 times higher in both Col‐0 and L*er* samples as compared to the expression of *RECQ4A*, *RECQ4B* and *FIGL1* (Figure 2b). These results suggest that the viral load in flower buds is high and is not the limiting factor in the silencing of meiotic genes.

### TRV‐*FIGL1* and TRV‐*RECQ4* markedly increase chiasma frequencies in Col‐0 *msh4* plants

The increased seed set in *msh4* plants after treatment with TRV‐*FIGL1* or TRV‐*RECQ4* suggests that the semi‐sterile *msh4* phenotype was rescued by elevated CO recombination. To test this, we fixed flower buds of incipient inflorescences of Col‐0 *msh4::*TRV‐*FIGL1* and *msh4*::TRV‐*RECQ4* plants at 4 and 5 weeks post‐infiltration to obtain meiocytes at different meiotic stages. As controls we also fixed inflorescences of Col‐0 wild‐type and Col‐0 *msh4* plants grown at the same time and under the same conditions. Meiosis in wild‐type *A. thaliana* typically shows the presence of five bivalents at diakinesis and/or metaphase I. For *msh4* plants the reported average number of chiasmata is 1.55 per meiosis and mostly univalents segregate during meiosis I (Higgins et al., 2004). On the other hand, *msh4 figl1* mutants and *msh4 recq4a recq4b* double mutants show a partial and complete restoration of bivalent formation, respectively (Girard et al., 2015; Séguéla‐Arnaud et al., 2015). The successful VIGS‐induced phenocopy of *figl1* and *recq4* mutant phenotypes in a *msh4* background would therefore be expected to show five bivalents and explain the restoration of fertility.

**Figure 3:**
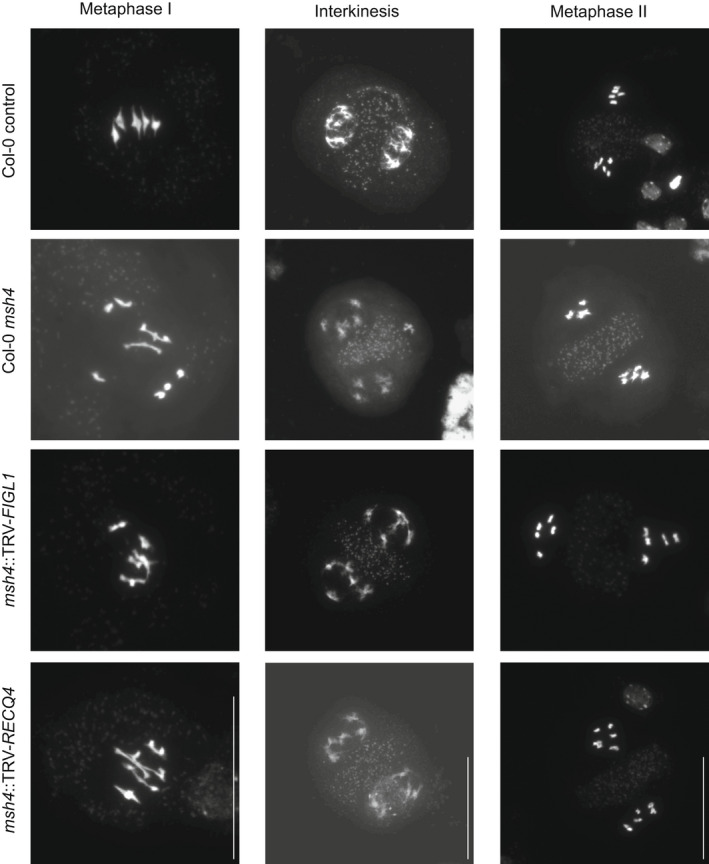
Cytological comparison of Col‐0 controls, Col‐0 *msh4*, *msh4*::TRV‐*FIGL1* and *msh4*::TRV‐*RECQ4* meiocytes. Meiocytes at metaphase I, interkinesis and metaphase II stages in a wild‐type background (Col‐0 control, first panel) show bivalent formation and balanced chromosome segregation. By contrast, images of chromosome spreads of *msh4* (second panel) depict achiasmatic chromosomes in metaphase I followed by irregular chromosome segregation. The images found in the third and fourth panels show that in both *msh4*::TRV‐*FIGL1* and *msh4*::TRV‐*RECQ4*, respectively, chiasma formation is restored in metaphase I, promoting balanced chromosome segregation, as observed in interkinesis and metaphase II. Scale bar, 50 μm.

In chromosome spreads of wild‐type Col‐0 we found five bivalents in 99.4% of cells, as only one cell with four bivalents was observed out of 178 cells (Table 1). Later meiotic stages in wild‐type meiosis showed no aberrations and all observed meiocytes (*n* = 266) were consistent with regular chromosome segregation: balanced numbers of chromosomes and chromatids throughout the first and second meiotic divisions and similar sized nuclei during interkinesis (two nuclei) and in meiotic tetrads (four nuclei) (Table 1, Figure 3). On the other hand, observations in *msh4* controls showed the severe consequences of reduced CO frequencies. Consistent with previously published results (Higgins et al., 2004), we found that *msh4* plants form chiasmata at a frequency of 1.27 per meiosis (*n* = 109), a large reduction in comparison to the average of 9.39 COs (*n* = 38) that were observed in wild‐type meiosis. Meiotic *msh4* cells show zero to three bivalents during prophase (*n* = 105) (Table 1). The reduced number of chiasmata in *msh4* plants results in typical aberrant phenotypes in later meiotic stages. After meiosis I, two, three or four nuclei were formed with uneven chromosome numbers or unequal sized nuclei (Figure 3). Once meiosis is complete, both tetrads and polyads were formed, often with nuclei strongly differing in size or chromosome number. Meiocytes (anaphase I to tetrad stage) showed segregation patterns consistent with regular segregation in 21% of the cells in *msh4* (*n* = 195) against 100% in wild‐type Col‐0 cells (*n* = 266) (Table 1).

**Table 1:** Observations from early to late meiotic stages in Col‐0 *msh4*, wild‐type Col‐0 control, Col‐0 *msh4*::TRV‐*FIGL1* and Col‐0 *msh4*::TRV‐*RECQ4*

Slide	Number of chiasmata	Number of bivalents						Anaphase I to tetrad stage		*n*
	Average (*n*)	5	4	3	2	1	0	Regular	Irregular	
Col‐0 *msh4* control										
Slide 01		‐	‐	‐	‐	‐	‐	14	44	58
Slide 02	0.86 (7)	‐	‐	‐	2	2	4	9	30	47
Slide 03	1.24 (37)	‐	‐	2	10	11	9	1	7	40
Slide 04	1.32 (65)	‐	‐	9	13	24	19	9	38	112
Slide 05		‐	‐	‐	‐	‐	‐	8	35	43
All the cells in Col‐0 *msh4* control	1.27 (SD ± 1.08; *n* = 109)	0	0	11	25	37	32	41	154	300
Col‐0 control										
Slide 01	‐	44	‐	‐	‐	‐	‐	‐	‐	44
Slide 02	9.50 (10)	14	‐	‐	‐	‐	‐	22	‐	36
Slide 03	9.26 (23)	57	1	‐	‐	‐	‐	161	‐	219
Slide 04	‐	‐	‐	‐	‐	‐	‐	47	‐	47
Slide 05	‐	‐	‐	‐	‐	‐	‐	34	‐	34
Slide 06	9.80 (5)	62	‐	‐	‐	‐	‐	2	‐	64
All the cells in Col‐0 control	9.39 (SD ± 0.86; *n* = 38)	177	1	0	0	0	0	266	0	444
Col‐0 *msh4*::TRV‐*FIGL1*										
Slide 01	7.86 (7)	13	1	‐	‐	‐	‐	46	0	60
Slide 02	1.07 (27)	‐	‐	2	6	11	8	‐	7	34
Slide 03	1.02 (41)	‐	1	1	9	13	17	15	11	67
Slide 04	3.00 (21)	1	0	14	3	1	2	28	6	55
Slide 05	8.71 (7)	23	1	‐	‐	‐	‐	62	‐	86
Slide 06	‐	‐	‐	‐	‐	‐	‐	6	‐	6
All the cells in Col‐0 msh4::TRV‐*FIGL1*	2.43 (SD ± 2.80; *n* = 103)	37	3	17	18	25	27	157	24	308
Col‐0 *msh4*::TRV‐*RECQ4*										
Slide 01	1.23 (35)	‐	‐	3	10	18	6	4	41	82
Slide 02	4.86 (7)	13	6	2	0	0	1	50	8	80
Slide 03	7.20 (15)	23	7	‐	‐	‐	‐	11	‐	41
Slide 04	1.40 (15)	0	0	2	3	6	4	4	31	50
All the cells in Col‐0 *msh4*::TRV‐*RECQ4*	2.86 (SD ± 2.77; *n* = 72)	36	13	7	13	24	11	69	80	253

The first column describes the genotype. The second column indicates the average number of chiasmata per sample. The number of bivalents per meiocyte ranges from zero to five and is given in columns 3 to 8. Bivalent numbers in cells of *msh4* plants were used as control range from zero to three, whereas all the wild‐type cells except one showed five bivalents. Columns 9 and 10 list cell numbers found from telophase I to the tetrad stage. Cells were scored as ‘regular’ if the counted chromosome and chromatid numbers per nucleus resembled those of a wild‐type meiosis and nuclei in interkinesis and tetrad cells were of equal size. Cells were scored as ‘irregular’ when chromosome and chromatid numbers suggested unbalanced segregation during the first meiotic division or when nuclear size in interkinesis or tetrad‐stage cells were of different size. The last column indicates the total number of cells analyzed (summing numbers from columns 3 to 10). The numbers of chiasmata in column 2 were estimated on a subset of the metaphase I cells listed in columns 3 to 8. Therefore, a separate *n* is given for column 2.

In some slides made using flower buds of *msh4*::TRV‐*FIGL1*, we found meiocytes for which the phenotype is consistent with *msh4* (Table 1). This is expected, as VIGS does not affect all cells (Calvo‐Baltanás et al., 2020). For example, slides 02 and 03 show average chiasma frequencies of 1.07 and 1.02, respectively, with univalent chromosomes and irregular chromosome segregation (Table 1). Note that more slides with low CO numbers were found, but not all were fully counted. Slide 04 shows an intermediate phenotype, in which 82% of late meiotic cells display a segregation pattern consistent with wild type and metaphase I cells with three chiasmata are overrepresented (a twofold increase compared to *msh4*). However, in slide 03, 58% of cells in late meiosis show regular chromosome segregation. Similarly, slides 01, 05 and 06 also resemble a wild‐type meiosis, with no apparent irregularities from metaphase I to the tetrad stage (*n* = 152) (Figure 3). Importantly, 94.7% of the cells in metaphase I found in slides 01 and 05 contain five bivalents (*n* = 38) with markedly higher chiasma frequencies (7.86 and 8.71 respectively) as compared to *msh4*. These results indicate that TRV‐*FIGL1* increased chiasma frequencies up to sixfold in comparison to our observations of *msh4* mutants (Table 1).

In slides obtained from individual flower buds of *msh4*::TRV‐*RECQ4*, we observed phenotypes consistent with a *msh4* phenotype, as well as cell populations showing an increase in chiasma frequencies and an apparent increase in chromosome segregation balance as compared to *msh4*, (in slides 01 and 04) (Table 1, Figure 3). In slide 03, we observed five or four bivalents in 77% and 13% of the cells, respectively (*n* = 30) and apparently regular chromosome segregation at late meiotic stages (*n* = 11) (Table 1, Figure 3). The average chiasma frequency was 7.2 per cell, which is 5.7 times higher than in the *msh4* mutant. Meiocytes in slide 02 showed an intermediate phenotype, where 57% of cells (*n* = 21) had five bivalents, with an average chiasma frequency of 4.86 per cell. Both slides 02 and 03 highlight the potential of TRV‐*RECQ4* to increase recombination in *msh4* mutant plants. Taken together, these data show that VIGS‐mediated silencing of *FIGL1* and *RECQ4* results in a significant increase of chiasma numbers in silenced cells (Table 1), which leads to the restoration of fertility in *msh4* mutants (Figure 2a).

### VIGS‐mediated silencing of *OSD1* generates unreduced gametes and tetraploid offspring

**Figure 4:**
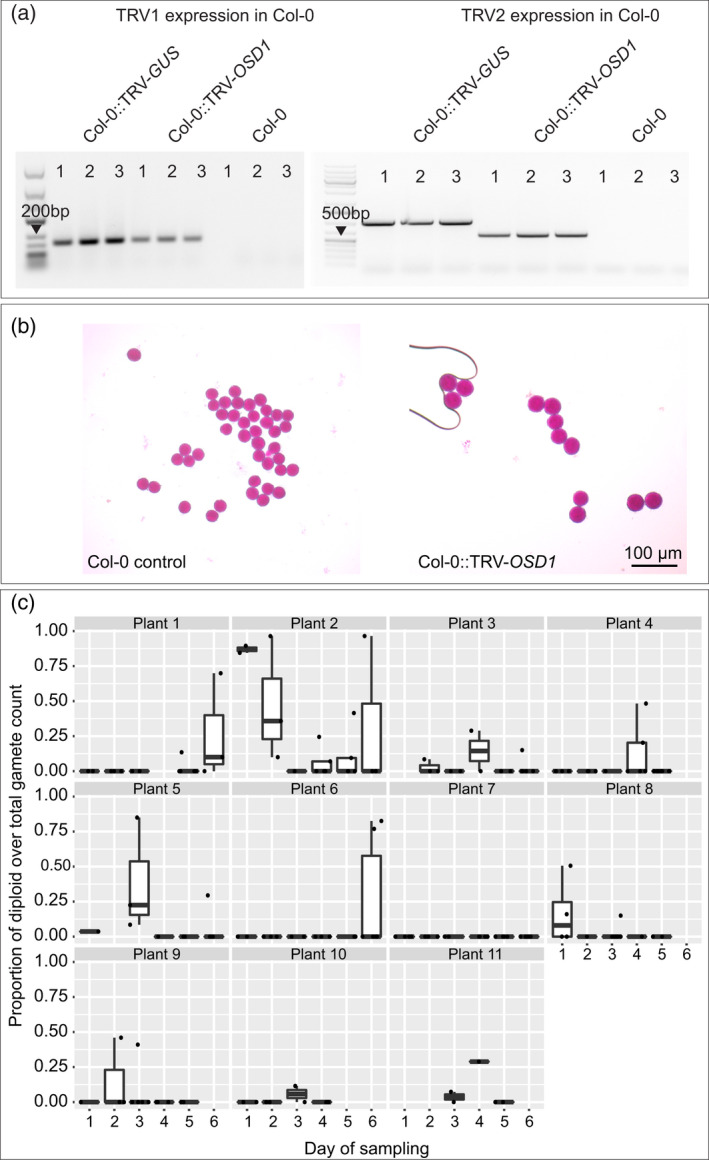
Diploid gamete production in Col‐0::TRV‐*OSD1* plants. (a) TRV1‐ and TRV2‐derived transcripts were detected in inflorescence samples of TRV‐*OSD1*‐inoculated Col‐0 plants, but not in Col‐0 controls. (b) Size comparison between haploid (left) and diploid gametes (right) was used as a visual proxy to assess the frequency of diploid pollen produced by Col‐0::TRV‐*OSD1* plants. Scale bar, 100 μm. (c) The proportion of large (diploid) gametes over the total number of gametes per plant and per day. Each dot represents a single flower. Note that some plants do not contain data for a certain day of sampling because no open flowers for that particular plant were present. [Colour figure can be viewed at wileyonlinelibrary.com]

To induce unreduced gamete formation and consequently the generation of polyploid offspring, we targeted *OSD1*, a gene essential for executing the second meiotic division (D'Erfurth et al., 2009). To develop a suitable VIGS construct, we selected a 250‐bp region spanning exons 1 and 2 of *OSD1* and cloned it into TRV, thus generating TRV‐*OSD1*. We initially decided to check the expression of TRV‐*OSD1* in treated plants. To this end, we infiltrated three Col‐0 plants with TRV‐*OSD1* and performed sqRT‐PCR on flower buds harvested 4 weeks post‐infiltration. TRV1 and TRV2 expression was observed in all infiltrated plants but not in Col‐0 non‐infiltrated controls (Figure 4a).

To evaluate if the expression of TRV‐*OSD1* in flower buds could induce large pollen (i.e., putative unreduced, diploid pollen; De Storme et al., 2013), we monitored pollen formation in 11 Col‐0 plants infiltrated with TRV‐*OSD1*. All open flowers of the 11 Col‐0::TRV‐*OSD1* were harvested for six consecutive days, starting at 27 days post‐infiltration, and we assessed the frequency of large pollen per flower, plant and day (Data S2). Additionally, we examined the pollen of three flowers of two non‐infiltrated Col‐0 plants as a reference. We observed that, while Col‐0 plants only produced uniform small pollen grains, Col‐0::TRV‐*OSD1* plants often produced larger pollen that likely corresponded to unreduced gamete production (Figure 4b, Data S2). Like our observations in plants treated with TRV‐*FIGL1* and TRV‐*RECQ4*, in which seed set in siliques showed high variation, here the fraction of large pollen in our samples was variable between flowers and between plants. (Figure 4c). For instance, Plant #2 produced two flowers on the first day of sampling, both with high, nearly identical rates of large pollen (84.5 and 85%). On the next day, three flowers opened with a noticeable variation of large pollen frequencies (10, 36 and 96%). On another plant (Plant #8), three flowers opened on the first day of sampling with 0, 16 and 50.6% of large pollen. The second day, one flower opened without (0%) large pollen, and the third day four flowers opened with 15%, 0%, 0% and 0% large pollen (Data S2, Figure 4c). However, despite the observed phenotypic variation, large pollen was present daily throughout the sampling time in the group of plants infiltrated with TRV‐*OSD1* (Figure 4c; Data S2).

The *osd1*−/− mutants in *A. thaliana* produce male and female diploid gametes and polyploid offspring upon self‐fertilization (D'Erfurth et al., 2009). For this reason, it is expected that also diploid plants infiltrated with TRV‐*OSD1* could yield tetraploid offspring. To verify that large pollen grains observed in Col‐0::TRV‐*OSD1* are indeed unreduced pollen and to test if both male and female meiosis produce unreduced gametes in response to TRV‐*OSD1*, we checked whether tetraploid offspring could be recovered from treated plants in four different *A. thaliana* accessions: Col‐0, L*er*, Est‐1 and Bor‐4. To this end, we first checked the sequence homology among the four *A. thaliana* accessions. Using the POLYMORPH tool (available at https://1001genomes.org/tools.html) we found that there are no single nucleotide polymorphisms (SNPs), insertions, or deletions in the coding sequence (CDS) of *OSD1* between Col‐0, L*er* and Bor‐4, but no data were available for Est‐1. This means that TRV‐*OSD1* should at least successfully trigger silencing in three accessions. We then infiltrated six Col‐0, 25 L*er*, 12 Est‐1 and 12 Bor‐4 plants with TRV‐*OSD1* and monitored the appearance of large pollen. Because the goal of the experiment was to obtain polyploid offspring from self‐fertilized plants, one or two anthers per flower were checked daily for the presence of large pollen. If a particular flower on a plant produced large pollen, this flower was marked as ‘positive’ and left to self‐fertilize. Once large pollen was first observed in a plant, phenotyping for that plant stopped. All the siliques formed before the day on which the plant was found positive were removed, while the flowers that formed afterwards remained on the plant for seed collection. Large pollen was observed in flowers of all Col‐0 plants infiltrated with TRV‐*OSD1*. For the other accessions, the percentages of plants showing a large‐pollen phenotype differed (L*er*: 32%; Est‐1: 8.33%; Bor‐4: 8.33%).

If TRV‐*OSD1* only affects male meiosis, one would expect to find a mixture of diploids and triploids among the offspring of treated plants in which large pollen were observed. If female meiosis was affected, tetraploids could be found among the offspring. Because tetraploid and triploid seeds derived from paternal genome excess are larger than diploid seeds (Ravi et al., 2008), we selected the largest seeds produced by six plants of Col‐0::TRV‐*OSD1*, one L*er*, one Est‐1 and one Bor‐4. These seeds were germinated, and the offspring ploidy was determined using a flow cytometer (see ‘Experimental Procedures’ section). We found that diploids, triploids and tetraploids were recovered for each accession. In addition to these selected seeds, we also grew a number of random seeds obtained from the same parental lines (Table 2). Selecting seeds on a size basis increased the frequency of finding tetraploid offspring for L*er* and Est‐1 and especially in Col‐0, but not in Bor‐4. In addition, we observed that the frequency of triploids is high in both Bor‐4 sets (Table 2). Because our samples were taken from the offspring of single plants from L*er*, Est‐1 and Bor‐4 accessions, it is difficult to make general statements on silencing efficiency differences between accessions. Nonetheless, these experiments demonstrate that the large pollen indeed corresponds to diploid pollen grains, as observed by the recovery of polyploid offspring in all accessions in response to TRV‐*OSD1*. Especially, the fact that tetraploid offspring was obtained from self‐fertilized plants highlights that unreduced gametes were also formed during female meiosis.

**Table 2:** Percentage of diploid, triploid and tetraploid offspring obtained from Col‐0, Est‐1, L*er* and Bor‐4 plants infiltrated with TRV‐*OSD1*

% 2*n* = 2*x*		% 2*n* = 3*x*	% 2*n* = 4*x*	Total number of plants
Size selection of seeds				
Col‐0	50.0 (*n* = 10)	10.0 (*n* = 2)	40.0 (*n* = 8)	20
Est‐1	0.0 (*n* = 0)	5.6 (*n* = 1)	94.4 (*n* = 17)	18
Ler	52.9 (*n* = 9)	0.0 (*n* = 0)	47.1 (*n* = 8)	17
Bor‐4	0.0 (*n* = 0)	95.0 (*n* = 19)	5.0 (*n* = 1)	20
Total seeds	25.7 (*n* = 19)	27.6 (*n* = 22)	46.6 (*n* = 34)	75
Random selection of seeds				
Col‐0	94.7 (*n* = 163)	4.70 (*n* = 9)	0.6 (*n* = 8)	180
Est‐1	37.5 (*n* = 6)	50.0 (*n* = 7)	12.5 (*n* = 12)	25
Ler	93.3 (*n* = 28)	0.0 (*n* = 0)	6.7 (*n* = 2)	30
Bor‐4	18.2 (*n* = 9)	72.7 (*n* = 26)	9.1 (*n* = 3)	38
Total seeds	60.9 (*n* = 206)	31.9 (*n* = 42)	7.2 (*n* = 25)	273

The percentages of diploids, triploids and tetraploids were evaluated in two different subsets for each accession. The first subset corresponds to selected seeds based on increased size which presumably leads to tetraploids or triploids originating from parental excess, as compared to smaller diploid seeds produced by diploid parents. The second subset was formed by randomly selecting seeds. For Col‐0, six plants were used to produce seeds, and only one plant was used for Est‐1, L*er* and Bor‐4.

### VIGS‐mediated silencing of *QRT2* generates pollen tetrads

**Figure 5:**
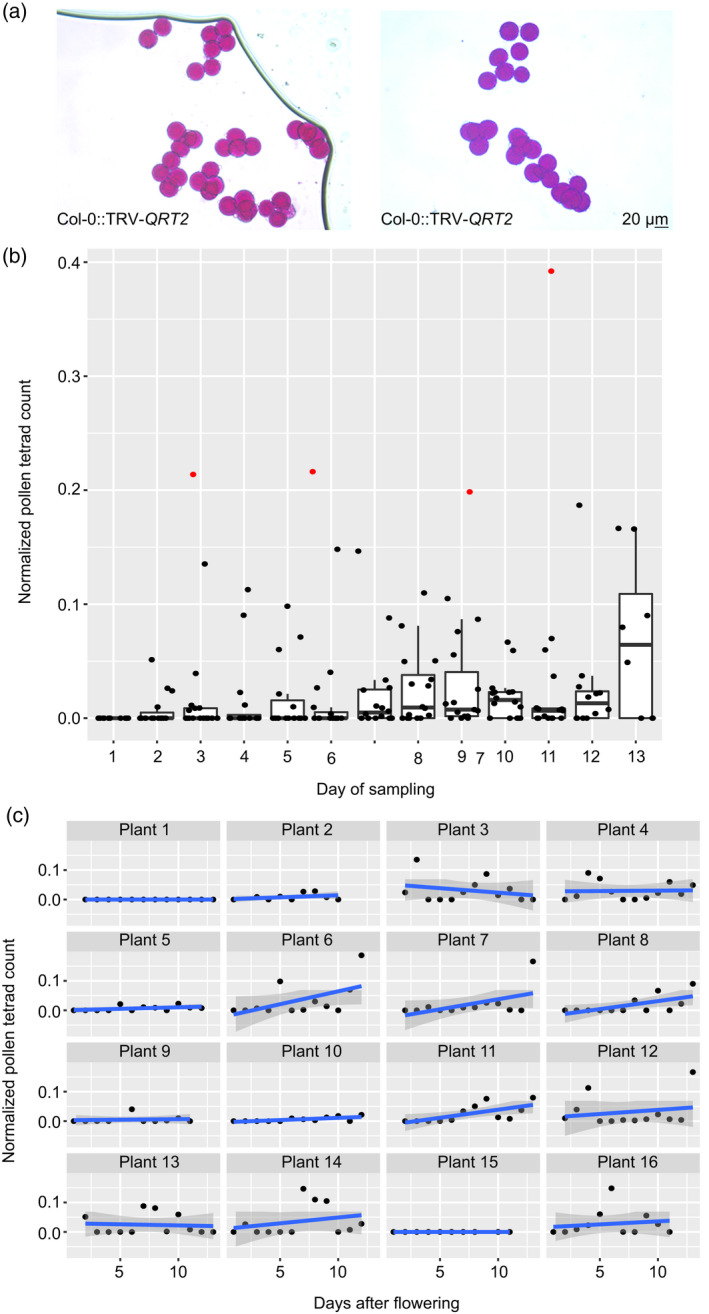
Pollen phenotyping in Col‐0::TRV‐*QRT2* plants. (a) Pollen tetrads produced by TRV‐*QRT2*‐infiltrated plants. Note that in both images, single pollen grains are found among pollen tetrads. Scale bar, 20 μm. (b) The normalized pollen tetrad count per day across the sampling period. Each dot represents a single plant (see ‘Experimental Procedures’ section). Red dots were identified as outliers by our model, and they were excluded from the analysis as per the protocol in Zuur et al. (2010). (c) The normalized number of tetrads per plant to the day in which each plant flowered within the sampling period. Note the large variation among plants. [Colour figure can be viewed at wileyonlinelibrary.com]

If VIGS were to be utilized to induce pollen tetrad formation, it would show that TRV can also be exploited to target genes active during microspore development. To our knowledge, the formation of pollen tetrad cannot be triggered by environmental conditions, and as such the VIGS‐mediated induction of pollen tetrads would also provide a robust phenotypic marker to assess silencing efficiency in pollen. To test if pollen tetrads could be induced, the TRV‐*QRT2* construct was created, harboring a 198‐bp fragment spanning *QRT2* exon 9. We infiltrated 16 Col‐0 plants and monitored the pollen phenotype in all open flowers for 13 consecutive days, starting on the day that the first plant flowered (i.e., day 1 of sampling). We observed that pollen tetrads were present from day 2 until sampling ended at day 13 (Figure 5, Data S3). Also, we observed that 14 out of 16 (87.5%) plants produced pollen tetrads on at least one day of sampling. However, like our previous experiments using TRV‐*FIGL1*, TRV‐*RECQ4* and TRV‐*OSD1*, the percentage of positive flowers for the silencing phenotype and the percentage of pollen tetrads per flower varied greatly within and between plants per day (Data S3, Figure 5).

We wondered whether there are specific factors that increase the likeliness of finding VIGS‐induced pollen tetrads among developing flowers. To this end we first identified several variables and checked if ‘number of tetrads (per day per plant)’ correlated to any other variables. Obvious positive correlations were only found with ‘number of positive flowers (per day per plant)’ and ‘total number of flowers (per day per plant)’ (Figure S7). From this correlation, we can only extrapolate that the more flowers are phenotyped, the more likely it is that positive flowers producing tetrads will be found. Since during later development more flowers open per day (i.e., on secondary branches), we specifically tested the hypothesis that more tetrads are produced towards the end of the sampling period. For this, we used a GLMM with normalized tetrad counts (see ‘Experimental Procedures’ section). This normalization allows us to fairly compare all the plants during the entire sampling period. Our analysis revealed a low correlation between ‘day of sampling’ and ‘normalized number of tetrads’ (*R* ^2^ = 0.116). Only one day of sampling (day 13) showed a significantly higher ‘number of tetrads’ (*P* < 0.001; Table S3, Figure 5). Without any data for later days, we cannot say if such a trend would continue over time. If there is any predictive value in the time of sampling, the effect is small on the first 13 days of flowering and of little practical value.

### Undetectable germline transmission of TRV1 and TRV2 in *A. thaliana*

If transient gene silencing is to be used to induce a phenotype in one generation only, TRV should not be transmitted to offspring. To confirm this, we tested for the presence of TRV1‐ and TRV2‐derived transcripts in the offspring of L*er* plants that were infiltrated with TRV‐*PDS*, of which we bulk‐harvested the seeds obtained from three visibly white siliques per plant (Figure S8). In addition, siliques from two non‐infiltrated controls were also harvested. We germinated the seeds obtained from the two L*er*::TRV‐*PDS* plants and observed that none of the seedlings showed whitening of leaves. From these seedlings, we randomly selected 15 from each mother plant and harvested them in groups of five. Each group of five was considered as a single sample. In total six samples (30 offspring) from treated plants and four samples (20 offspring) from untreated plants were used to conduct sqRT‐PCR analysis. We detected the presence of neither TRV1 nor TRV2 in any of the samples, while TRV1 and TRV2 were detected in two positive control samples obtained from inflorescences of L*er*::TRV‐*mCherry*. The presence of the reference gene *AT3G47060* was detected in all the samples except for the negative control (Figure S8). These results support the assumption that TRV‐VIGS assays yield TRV‐free offspring in *A. thaliana*.

## DISCUSSION

Previously, it was reported that class I CO formation could be reduced by targeting MSH5 in *A. thaliana* using VIGS (Calvo‐Baltanás et al., 2020). In this study, we show that class II CO formation can be upregulated by silencing *RECQ4A/RECQ4B* and *FIGL1*, which lead to a sixfold increase in chiasma numbers in a *msh4* mutant background. We also showed that the execution of the second meiotic division can be bypassed to obtain unreduced gametes and polyploid offspring by silencing *OSD1*. Lastly, targeting *QRT2* resulted in the formation of pollen tetrads for at least 12 consecutive days in a group of treated plants. Importantly, these experiments prove that both male and female meiosis can be targeted (*OSD1*) and that two gene copies can be downregulated by the same construct (*RECQ4A/RECQ4B* in Col‐0 *msh4*). These data also demonstrate that the VIGS‐mediated silencing phenotype for *OSD1*, *RECQ4* and *FIGL1* can be reproduced in different accessions.

In wheat (*Triticum aestivum*), VIGS has been used to target early meiotic processes like homologous and homeologous recombination through *DMC1*, *XRCC2* and *C‐Ph1* (Bennypaul et al., 2012; Bhullar et al., 2014; Desjardins et al., 2020; Raz et al., 2021) or the positioning of recombination events by targeting the genes *MET1* or *DDM1* (Raz et al., 2021). Raz et al. (2021) observed no effect of silencing of *FANCM*, a known negative regulator of class II CO. Our data, however, show that targeting this pathway with VIGS is possible at least in *A. thaliana*. All combined, VIGS emerges as a generic tool to control gene expression throughout meiosis and early pollen formation, not only in the monocot wheat, but also in the dicot *A. thaliana*, with potential to be rapidly translated to other species.

### VIGS‐induced phenotypes in different *A. thaliana* genetic backgrounds

Targeting *OSD1* to generate polyploid offspring in four different accessions illustrates the potential of VIGS to modify meiosis in different genetic backgrounds. Moreover, it was shown that the constructs TRV‐*RECQ4* and TRV‐*FIGL1* cause an increase of chiasma frequencies in both L*er* and Col‐0 backgrounds, leading to the restoration of fertility in *msh4* mutant lines. Interestingly, during these experiments we observed noticeably higher seed sets in L*er* than in Col‐0 plants, the precise cause of which remains unclear. In Col‐0, *RECQ4A* and *RECQ4B* need to be simultaneously inactivated to achieve an increase in CO frequencies, as opposed to just *RECQ4A* in L*er* (Séguéla‐Arnaud et al., 2015). However, a lower silencing efficiency due to the presence of two functional *RECQ4* genes in Col‐0 *msh4* is unlikely to cause the higher seed set in L*er*, since we observed that the silencing of *FIGL1*, a single gene, also results in a higher seed set increase in L*er msh4* as compared to Col‐0 *msh4*. We therefore hypothesize that this distinct phenotype might be caused by genetic differences between L*er* and Col‐0. In addition to the differential phenotype for the particular trait ‘seed set’ between the two accessions, it has been noted before that the impact of *figl1* and *recq4* mutations on seed set and pollen viability is background‐dependent, as for example described for Col‐0 and Col‐0‐L*er* hybrid backgrounds (Fernandes et al., 2017). Col and L*er* also differ in a natural genetic polymorphism in the gene *HEI10*, which is known to slightly increase the number of class I COs in L*er* (Chelysheva et al., 2012; Ziolkowski et al., 2017). The possibility of uneven TRV viral expression between accessions seems also unlikely, since there were no significant differences in the expression of TRV2 in inflorescences between Col‐0 and L*er*. Interestingly, not only the viral load was equal for both accessions, but it was about 32 times higher than the endogenous expression of the target genes *FIGL1* and *RECQ4A/RECQ4B*. High expression of TRV in inflorescences indicates that transmission to the meristem is not restricted. However, there is a disproportionate low number of gametes affected by the silencing, which indicates that other mechanisms may affect the transmission of TRV or derived siRNAs to meiocytes.

### Opportunities of VIGS for meiosis in *A. thaliana* and crop species

There is a rising interest in the transient modification of meiosis in crops, and several reports in wheat have shown the use of VIGS in modifying meiotic recombination (Bennypaul et al., 2012; Bhullar et al., 2014; Desjardins et al., 2020; Raz et al., 2021). Based on these results and the data presented here, it is possible to delineate various applications for which VIGS could be utilized. An obvious possibility could lie in increasing meiotic recombination frequencies to produce high‐resolution mapping populations in *A. thaliana* and crops. Mutants of *RECQ4* led to an increase of recombination frequencies in pea (*Pisum sativum*), tomato (*Solanum lycopersicum*) and rice (Mieulet et al., 2018) and a mutation in *FANCM* results in an about twofold increase in recombination in hybrids of rice and pea but not in tomato (Mieulet et al., 2018). The *fancm* mutants in *Brassica rapa* and *Brassica napus* also showed a significant increase in recombination frequencies, but the effect on hybrids has yet to be assessed (Blary et al., 2018). Interestingly, the reported mutants of *FIGL1* in crops do not set seed (Zhang, Zhang, et al., 2017; Mieulet et al., 2018), thus making it impossible to generate mapping populations. Constructs targeting *RECQ4* or *FANCM* are therefore the more likely candidates to exploit high recombination frequencies in a wider range of species, especially in slow cycling plants. A genetic screening with VIGS can avoid the laborious and lengthy production of stable mutations that do not lead to the desired phenotype, like *fancm* mutants in tomato or those that lead to sterility, such as observed in *figl1* mutants of pea, rice and tomato. The studies in wheat elegantly demonstrated the benefits of using VIGS in a polyploid crop, in which generating stable null mutants is even more challenging. In addition, stable mutations in genes essential to somatic DNA repair like *RECQ4* (Hartung et al., 2007; Kwon et al., 2013) could lead to genomic instability over generations, and in this regard, VIGS could represent a much more desirable alternative.

The use of VIGS for generating triploid and tetraploid offspring through SDR gametes is straightforward. VIGS‐mediated unreduced gamete production may be preferred over other methods like colchicine, nitrous oxide (N_2_O) and heat or cold shock (Bishnoi et al., 2000; De Storme et al., 2012; Dvorak et al., 1973; Forster et al., 2007; Wang et al., 2017; Younis et al., 2014) for which adverse side effects such as genome instability, mitotic abnormalities and changes in chromosome structure have been reported (Al‐Khayri et al., 2016; Kundu & Ray, 2017; Rodriguez et al., 2001). Though not tested, our experiments and those of others suggest that the downregulation of genes acting during the first meiotic division like *JASON* (*JAS*) or *PARALLEL SPINDLE 1* (*P1*) can also be induced (D'Erfurth et al., 2008; De Storme & Geelen, 2011), thus allowing the generation of first division restitution (FDR) gametes.

With VIGS well developed for modifying inheritance in wheat and *A. thaliana*, there is potential to investigate its use to modulate meiosis in other species. For example, TRV‐VIGS is available for tomato (Brigneti et al., 2004; Liu, Schiff, & Dinesh‐Kumar et al., 2002; Meng et al., 2016), poppy (*Papaver somniferum*) (Hileman et al., 2005), maize (Zhang, Yu, et al., 2017), spinach (*Spinacia oleracea*) (Lee et al., 2017) and *Cannabis sativa* (Schachtsiek et al., 2019). The wheat VIGS system based on the Barley stripe mosaic virus was successfully used for a large genetic screen to identify the causal gene preventing homoeologous recombination, *C‐Ph1*, among 26 candidate genes (Bhullar et al., 2014) and to silence the meiotic genes *DMC1* and *XRCC2* (Bennypaul et al., 2012; Desjardins et al., 2020; Raz et al., 2021). The VIGS system obtained from the engineered Rice tungro bacilliform virus (RTBV) has achieved 40–80% silencing efficiency in rice (Kant & Dasgupta, 2017). The bipartite VIGS vector based on the RNA Pea early browning virus (PEBV) caused up to 94% silencing of *PDS* in vegetative tissues and genes involved in floral development were successfully downregulated, leading to mutant floral phenotypes (Constantin et al., 2004). Later, another study in pea showed that the VIGS system developed from the Bean pod mottle virus (BPMV) caused efficient silencing in 36 different genotypes, with viral expression observed in flowers in several of these lines (Meziadi et al., 2016). All in all, there seem to be ample opportunities to develop VIGS systems to transiently induce any meiotic and gamete‐specific phenotype, not only in model organisms but also in crop species.

## EXPERIMENTAL PROCEDURES

### Plant growth and infiltration with VIGS constructs

All the plants were germinated on 1/2 MS + vitamins plates with 1% sucrose and transferred to potting soil 7–10 days after germination. Once on soil, plants were grown in a Percival growth chamber under a 16/8‐h light/dark cycle (21°C/18°C) and 50–60% relative humidity. Plants for all the experiments were infiltrated 3 weeks post‐germination, except for those used in dPCR experiments and to assess transgenerational TRV transmission by sqRT‐PCR, which were infiltrated 2 weeks post‐germination. The bacteria incubation and leaf‐infiltration protocols were previously described (Nimchuk et al., 2000; Vaghchhipawala et al., 2011) with one of the vectors of choice (TRV‐*FIGL1*, TRV‐*RECQ4*, TRV‐*OSD1*, TRV‐*QRT2*, TRV‐*GUS*, TRV‐*mCherry*, TRV‐*PDS*) mixed with TRV1 (Burch‐Smith et al., 2006) in a 1:1 ratio. For all the experiments, four Col‐0 plants were always infiltrated with TRV‐*PDS* as positive controls for successful treatment.

### VIGS constructs

To select an appropriate CDS region of the target genes, we used the online tool SGN VIGS to detect possible off‐targets of homologous sequences between 150 and 250 bp. To generate TRV‐*FIGL1*, a 169‐bp CDS of *FIGL1* of the Col‐0 reference genome was selected. The construct TRV‐*RECQ4* contains a 240‐bp fragment cloned into TRV2. This sequence is 100% identical to the *RECQ4A* CDS and shares 88% sequence identity with the *RECQ4B* CDS, both in Col‐0. The donor sequences for the constructs targeting *FIGL1* and *RECQ4* were commercially synthesized by Thermofisher Scientific (Waltham, MA, USA) as a single synthetic DNA fragment which was delivered in a pMK‐RQ plasmid. Restriction sites were introduced in the primers used for amplification of both fragments using the donor plasmid as template (Table S4). To clone the VIGS constructs targeting *OSD1* and *QRT2*, RNA was first extracted from Col‐0 flower buds using TRIzol™ reagent (Thermofisher) and cDNA was synthesized through a reverse‐transcription reaction, following the manufacturer's protocol. The generated cDNA was used as a template to amplify a 250‐bp fragment of *OSD1* and a 198‐bp fragment of *QRT2* using primers that introduced restriction sites (Table S4). To generate TRV‐*mCherry*, a 245‐bp fragment obtained from pICSL30003 (Engler et al., 2014) was subcloned into TRV2 (Table S4). All fragments were cloned into TRV2 in either sense or antisense orientation following a classical digestion–ligation method using FastDigest enzymes and T4 ligase from Thermofisher scientific (Table S4). All VIGS constructs were verified by Sanger sequencing using the TRV2_seq primer (Table S4). *Agrobacterium tumefaciens* GV3101 transformed with each construct was selected using rifampicin and kanamycin, and successful transformation was confirmed by colony PCR.

### Seed set evaluation

Col‐0 and L*er* plants used in experiments with TRV‐*FIGL1* and TRV‐*RECQ4* were grown and left to set seed. Seed set evaluation in Col‐0::TRV‐*FIGL1* and Col‐0::TRV‐*RECQ4* plants was done by collecting 10 siliques from the first 25–30 formed siliques on the main stem in each plant. In the case of *msh4* Col‐0 and *msh4* L*er* plants infiltrated with both constructs, we collected the longest siliques present on the main stem. Siliques were cut open and putative viable seeds were identified by their bright green coloration and regular shape (as observed in Col‐0 wild‐type siliques). Aborted seeds were identified as desiccated seeds, unusual small seeds and/or seeds that displayed a pale coloration.

### Pollen phenotyping

Pollen of individual flowers from a total of 11 plants infiltrated with TRV‐*OSD1* and from 16 plants infiltrated with TRV‐*QRT2* were checked by dipping and shaking each flower vigorously onto 13 μl of Alexander staining solution (Peterson et al., 2010) to release the pollen. Pollen was then evaluated under a Zeiss light microscope. For phenotypic evaluation of pollen produced by TRV‐*OSD1*‐infiltrated plants, all newly opened flowers were sampled every day for all plants throughout six consecutive days. The number of unreduced (large) pollen was counted in a total of 200 pollen grains whenever possible (Data S2). Pollen from three flowers of two non‐infiltrated Col‐0 plants grown alongside were used as a negative control during the experiment to check for the presence of large or aborted pollen as a possible sign of environmental stress and as reference for haploid pollen size.

In the set of 16 plants infiltrated with TRV‐*QRT2*, we harvested a total of 1154 flowers over 13 consecutive days. A maximum of 18 random flowers – from the main stem and the lateral branches – were harvested each day per plant. The number of pollen tetrads in a total of 250 pollen grains per flower was counted when possible (Data S3). Each pollen tetrad was counted as four pollen grains. Three non‐infiltrated Col‐0 plants were used as controls by using pollen from two flowers per plant every day during the 13‐day sampling period.

### Selection of polyploid offspring in Col‐0, L*er*, Est‐1 and Bor‐4

Ploidy of offspring derived from plants producing unreduced gametes of each accession was confirmed by flow cytometry analysis using 0.5 cm fresh leaf samples and a CyFlow® Ploidy Analyzer from Partec. As controls for diploid and tetraploid offspring, we used the leaf material from a Col‐0 diploid plant and a tetraploid plant selected from the self‐fertilized progeny of a Col‐0 *osd1* (*osd1‐3*; Heyman et al., 2011) heterozygous mutant.

### sqRT‐PCR and dPCR expression analysis of TRV1 and TRV2 in inflorescences

To check for the expression of TRV1 and the different TRV2 constructs, flower buds from infiltrated plants of Col‐0, L*er*, *msh4* Col‐0 and *msh4* L*er* were harvested at either 21 or 28 days post‐infiltration, when the incipient main inflorescence developed. RNA was extracted from flower buds using TRIzol™ reagent (Thermo Scientific) or one‐step RNA reagent (Biobasic, Singapore). A total of 1 μg RNA per sample was treated with Thermo Scientific DNase I, RNase‐free and used to obtain cDNA through reverse transcription using either the Thermo Scientific RevertAid First Strand cDNA synthesis kit or the Maxima First Strand cDNA Synthesis Kit following the manufacturers' protocols. Of the obtained cDNA, 1 μl was used as template for a 10‐μl RT‐PCR reaction using either TAQ or Primer Star Max following the conditions for TAQ/Primer Star Max: Initial denaturation (5/2 min at 95/98°C), followed by 22 cycles of denaturation (30/10 sec at 95/98°C), primer annealing (30/5 sec at 55°C) and elongation (1 min/20 sec at 72°C) and a final elongation step (10/2 min at 72°C). The quality of the cDNA for each sample was assessed by amplifying a 80‐bp fragment of *At3G47060* or a 2427‐bp fragment of *PDS5* (Table S4).

To detect TRV1 and TRV2 in *msh4* Col‐0 infiltrated with TRV‐*FIGL1*, TRV‐*RECQ4* and TRV‐*GUS* and in Col‐0 infiltrated with TRV‐*GUS* and TRV‐*OSD1*, we used the primers RT_TRV1_Fw and RT_TRV1_Rw (Calvo‐Baltanás et al., 2020) and TRV2_Fw and TRV2_Rw (Hileman et al., 2005) (Table S4). To check for the expression of TRV1 and TRV2 in Col‐0 and L*er* plants infiltrated with TRV‐*mCherry* and the offspring of L*er* plants infiltrated with TRV‐*PDS*, we used the primers TRV1_Fw and TRV1_Rw and TRV2_Fw and TRV2_Rw (Hileman et al., 2005) (Table S4).

### dPCR expression analysis of TRV‐*mCherry* , *FIGL1* , *RECQ4A* and *RECQ4B* in Col‐0 and L*er* inflorescences

We measured the absolute expression level of TRV2‐*mCherry*, endogenous *FIGL1*, *RECQ4A*, *RECQ4B*, *At4G26410* and *At3G47060* in every sample, L*er*::TRV‐*mCherry* (*n* = 6) and Col‐0::TRV‐*mCherry* (*n* = 6), using dPCR technology. The reference gene *At3G47060* encodes a chloroplast‐localized FtsH protein and its expression is stable in the shoot apex (Liu et al., 2010; Sakamoto et al., 2003). In addition, *At4G26410* is often used as a reference gene for different developmental stages in plants that are also subjected to different abiotic and biotic stress conditions (Hong et al., 2010; Kudo et al., 2016). Because we only aimed to detect one target per reaction, we set a total of six reactions per sample to assess the expression of the six target genes independently. For this, 0.5 μl of cDNA per sample was used as template for a 12‐μl dPCR reaction volume in EVAGREEN master mix (QIAGEN, Hilden, Germany). All the reactions were run in a QIAcuity One, 5plex device (QIAGEN) using a single 8.5 K 96‐well nanoplate (QIAGEN). The PCR conditions were the following: initial heat activation (2 min at 95°C), followed by 40 cycles of denaturation (15 sec at 95°C) and annealing/extension (30 sec at 60°C). The plate was imaged with an exposure time of 300 msec using the green channel (gain = 0). The primers used to check for the expression of *RECQ4A*, *RECQ4B*, *FIGL1*, TRV‐*mCherry*, *AT4G26410* and *ATG3G47060* can be found in Table S4.

### Cytology

Flower buds from non‐infiltrated Col‐0 and Col‐0 *msh4* plants and from Col‐0 *msh4* plants infiltrated with TRV‐*FIGL1* and TRV‐*RECQ4* were bulk‐harvested (according to the specific treatment) at 4 and 5 weeks post‐infiltration, fixed in Carnoy fixative (3:1 glacial acetic acid [HAc]:99.8% EtOH) and kept overnight at 4°C. Flower buds were then washed twice with 70% EtOH dissolved in water and stored at 4°C until further use. To make meiotic chromosome spreads we followed the protocol previously described in Ross et al. (1996). Chromosome spreads were stained with 4′,6‐diamidino‐2‐phenylindole (DAPI) in Vectashield and analyzed using a Zeiss microscope equipped with epifluorescence optics.

### Statistical analysis

For all phenotypes our datasets consist of groups of plants with multiple observations taken from each individual. Consequently, observations made to the same plant are not only affected by the explanatory variables but may also be confounded by plant‐specific effects. This non‐independence prevents us from using commonly employed statistical techniques (such as pairwise comparisons like the *t*‐test) for data analysis. Instead, we opted for linear mixed‐effect models and their variants to account for this non‐independence of observations. In mixed‐effect models, one can specify the plant ID for a group of observations; the model uses this information to generate a linear regression with a unique intercept for each plant ID. Simply, the intercept will be the value of the response variable when continuous explanatory variables are equal to zero and when the categorical variables take the value of the reference group. The visual representation of these models can be found on Github (https://github.com/CherWeiYuan/VIGS_Meiosis_A._thaliana).

To ensure that mixed‐effect models are reliable, we performed exploratory data analysis to filter the data and carried out a series of checks in accordance with available protocols (Zuur & Leno, 2016; Zuur et al., 2010). Datasets were first screened with boxplots and Cook's distance to remove outliers. Next, we transformed the response variable if it did not follow a normal distribution or was heteroscedastic. Collinear explanatory variables were removed by the variance inflation factor (VIF). Homoscedasticity of the model was checked with residual plots. All statistics were performed on base R (R Core Team, 2020) on RStudio (RStudio Team, 2020). Packages used were ggplot, lattice, DHARMa, car, glmmTMB, tidyverse, caret, plyr, lme4, nlme, dplyr, rcompanion and effects as denoted in the R script uploaded to https://github.com/CherWeiYuan/VIGS_Meiosis_A._thaliana.

All our linear regression (binomial/negative binomial GLMM with or without accounting for zero inflation) were tested with k‐fold validation by training the model with a randomized 90% of the dataset and testing it 10 times against the remaining 10%. This simulation produced an *R* ^2^ value which is the fraction of the total variance within the dataset explained by the model. A higher *R* ^2^ means that the explanatory variables are good predictors of the dataset, which in turn indicates that these variables are correlated with the response variable.

#### Odds of obtaining viable seeds in Col‐0 plants treated with TRV‐*FIGL1* , TRV‐*RECQ4* and TRV‐*GUS* (GLMM)

We investigated the odds of obtaining viable seed counts per silique between treated Col‐0 plants and controls. The data were filtered by removing outliers with extremely high or low viable or aborted seed counts. As our response variable is probabilistic, we opted for binomial regression: The odds of viable over aborted seeds per silique (continuous variable) is a function of treatment (categorical with four levels: TRV‐*RECQ4*, TRV‐*FIGL1*, TRV‐*GUS* and Col‐0 untreated). In this dataset, each observation corresponds to one silique. As 10 siliques were obtained per plant, the assumption that siliques are independent from one another was violated. To resolve this issue, we introduced plant ID as a random intercept in our models. Each plant is given a different intercept to represent possible differences in viable seed production. Consequently, we built up a binomial GLMM represented by the following equation:

$$\text{odds of viable over aborted seeds}\;\mathrm{per}\;\text{silique}∼\mathrm{TRV}\;\text{treatment}+\left(1|\;\text{plant}\;\mathrm{ID}\right).$$

#### Rescue of semi‐sterility of *msh4* mutant lines

We studied the variable ‘well‐developed seeds per silique’ as a proxy for viable seeds produced by *msh4* mutants in Col‐0 and L*er* in non‐treated controls and after treatment with TRV‐*RECQ4*, TRV‐*FIGL1* and TRV‐*GUS*. The response variable consists of seed counts, which are never negative (suitably modeled by Poisson or negative binomial regression), and we used mixed‐effect modeling to account for plant‐specific effects. Therefore, we built a negative binomial GLMM. The number of viable seeds per silique is expressed as a function of treatment type (categorical with four levels: TRV‐*RECQ4*, TRV‐*FIGL1*, TRV‐*GUS* and untreated Col‐0 *msh4*) and accessions (Col‐0 and L*er*):

$$\text{viable seeds}\;\mathrm{per}\;\text{silique}∼\text{treatment type}+\text{accession}+\left(1|\;\text{plant}\;\mathrm{ID}\right).$$

#### Pollen tetrad counts over time

For this dataset, we defined day 1 as the day when we observed the first open flower in each plant. Pollen tetrad number per day and per plant was calculated as the proportion of pollen tetrads on a day over the total number of tetrads produced by that plant across the sampling period (13 days). In addition, tetrad count is positively correlated with the number of positive flowers and total number of flowers produced per plant and per day, thus we need to account for differences in flower productivity. Therefore, our response variable, pollen tetrad count, is normalized as follows:

$$Y=\frac{\;\text{tetrads}\;\mathrm{on}\;\mathrm{day}\;x\;\text{for plant}\;y}{\text{tetrads produced}\;\mathrm{by}\;\text{plant}\;y\;\text{across}\;\mathrm{all}\;\text{days}}*\frac{\text{positive flowers}\;\mathrm{on}\;\mathrm{day}\;x}{\left(\text{total flowers}\;\mathrm{on}\;\mathrm{day}\;x\right)+1}.$$

The response variable (number of tetrads) does not occur as negative values, and hence they are better represented by the Poisson or negative binomial distribution. To fit the data to these regression models (where the response variable takes either zero or positive integers), we transformed *Y* by multiplying it by 10^3^ before rounding it into the nearest integer. Since 52.4% of the observations show zero tetrad counts, the data were fitted to a zero‐inflated model. After removing two outliers as they represent extremely rare occurrences in which tetrad production spiked (Plant #9 on days 6 and 13), we fitted the data to a negative binomial zero‐inflated GLMM with plant ID as a dependence structure:

$$Y∼\mathrm{day}+\left(1|\;\text{plant}\;\mathrm{ID}\right).$$

## ACCESSION NUMBERS

Plant accessions Col‐0 (ABRC CS76113), L*er* (L*er*‐1; ABRC CS76164), Bor‐4 (ABRC CS76100) and Est‐1 (ABRC CS76127) used in this study were selected from the collection of natural accessions belonging to the Hapmap panel (Baxter et al., 2010; Li et al., 2010; Horton et al., 2012). The accession L*er*‐0 (ABRC CS24238) was used for sqRT‐PCR and dPCR experiments. The *msh4‐1* mutant in a Col‐0 background (Salk_136296) and genotyping primers were described by Higgins et al., 2004. The *msh4* mutant in a L*er* background (CSHL‐GT14269) was provided by R. Mercier (Institut Jean‐Pierre Bourgin, INRA, AgroParisTech, CNRS, Université Paris‐Saclay, Versailles, France). The VIGS vector TRV‐*GUS* used as a negative control was provided by Laurens Deurhof (Laboratory of Phytopathology, Wageningen University, the Netherlands), TRV1 and TRV2 vectors can be retrieved from ABRC under stock numbers CD3‐1039 and CD3‐1040, respectively. The vector TRV‐*PDS* can be found under stock number CDR‐1047.

## AUTHOR CONTRIBUTIONS

EW and VC‐B conceptualized the research; AS, EC and EW were involved in supervision and responsible for funding acquisition; cloning of VIGS constructs was done by VC‐B; VC‐B, NS and EW performed VIGS and phenotyping experiments; VC‐B and JDJ‐B performed expression analyses and phenotyping experiments; EW performed cytogenetic analyses; VC‐B, JDJ‐B, WYC and EW processed and interpreted experimental data, designed the figures and drafted the manuscript with the help of NS, EC and AS. All authors discussed the results and commented on the manuscript.

## CONFLICT OF INTEREST

The authors declare no conflict of interest.

## Supporting information


**Figure S1.** sqRT‐PCR analyses in infiltrated and non‐infiltrated plants to detect TRV1 and TRV2 expression in flower buds of Col‐0 and L*er* plants infiltrated with TRV‐*FIGL1*, TRV‐*RECQ4*, TRV‐*GUS* and TRV‐*mCherry* but not in untreated controls.
**Figure S2.** Col‐0 plants infiltrated with TRV‐*RECQ4*, TRV‐*FIGL1* and TRV‐*GUS* do not display shortened siliques or differences in the number of well‐developed seeds as compared to Col‐0 non‐infiltrated controls.
**Figure S3.** Col‐0 plants infiltrated with TRV‐*GUS*, TRV‐*FIGL1* and TRV‐*RECQ4* do not display significant differences in the number of viable or aborted seeds per silique in comparison to non‐infiltrated Col‐0 controls.
**Figure S4.** Model fit of negative binomial GLMM for Col‐0 *msh4* shows differences in the predicted value for the variable ‘viable seeds per silique’ in infiltrated plants with TRV‐*RECQ4* and TRV‐*FIGL1* as compared to controls.
**Figure S5.** Phenotypic comparison of L*er msh4* plants infiltrated with TRV‐*RECQ4* and TRV‐*FIGL1* reveals an increase in silique length and seed numbers as compared to L*er msh4* controls and L*er*::TRV‐*GUS*.
**Figure S6.** Model fit of negative binomial GLMM for L*er msh4* shows differences in the predicted value for the variable ‘viable seeds per silique’ in infiltrated plants with TRV‐*RECQ4* and TRV‐*FIGL1* as compared to controls.
**Figure S7.** Pair‐plot shows a direct correlation between the variable ‘number of tetrads’ per day of sampling and per individual with the variables ‘number of positive flowers’ and ‘total number of flowers’ in Col‐0 plants treated with TRV‐*QRT2* and monitored for 13 consecutive days.
**Figure S8.** Photobleaching in L*er*::TRV‐*PDS* plants and absence of TRV1 and TRV2 expression in *A. thaliana* offspring obtained from treated plants.

**Table S1.** Model summary for binomial (logit) GLMM: odds of viable over aborted seeds per silique ~ treatment + (1| plant ID) for non‐infiltrated Col‐0 and infiltrated Col‐0 with TRV‐*RECQ4*, TRV‐*FIGL1* and TRV‐*GUS*.
**Table S2.** Model summary for negative binomial GLMM: viable seeds per silique ~ treatment + (1| plant ID) for Col‐0 and L*er msh4* mutants.
**Table S3.** Model summary for negative binomial GLMM for normalized tetrad counts from *QRT2* knock‐down: normalized tetrads ~ day + (1| plant ID).
**Table S4.** List of primers used to create and confirm by Sanger sequencing the generated VIGS constructs and assess TRV1 and TRV2 expression by semi‐quantitative real‐time PCR (sqRT‐PCR) and digital PCR (dPCR).

**Data S1.** Seed set numbers in Col‐0 wild‐type, Col‐0 *msh4* and L*er msh4* plants non‐infiltrated and infiltrated with TRV‐*RECQ4*, TRV‐*FIGL1* and TRV‐*GUS*. Transcript expression levels of TRV‐*mCherry* and endogenous *FIGL1*, *RECQ4A* and *RECQ4B* in inflorescence samples of Col‐0::TRV‐*mCherry* and L*er*::TRV‐*mCherry*.

**Data S2.** Pollen phenotyping data obtained from Col‐0::TRV‐*OSD1* plants during six consecutive days.

**Data S3.** Number of pollen tetrads produced by Col‐0::TRV‐*QRT2* plants per flower and per plant over a total of 13 sampling days.

## Data Availability

All relevant data can be found within the manuscript and its supporting materials.
