# Supplementary material for: Knock‐down of gene expression throughout meiosis and pollen formation by virus‐induced gene silencing in Arabidopsis thaliana
Source: Plant J. 2022 Jun 18;111(1):19–37. doi: 10.1111/tpj.15733 (PMC9543169; doi:10.1111/tpj.15733)
Supplement: Supplementary file 1 — Figure S1. sqRT‐PCR analyses in infiltrated and non‐infiltrated plants to detect TRV1 and TRV2 expression in flower buds of Col‐0 and Ler plants infiltrated with TRV‐FIGL1, TRV‐RECQ4, TRV‐GUS and TRV‐mCherry but not in untreated controls. Figure S2. Col‐0 plants infiltrated with TRV‐RECQ4, TRV‐FIGL1 and TRV‐GUS do not display shortened siliques or differences in the number of well‐developed seeds as compared to Col‐0 non‐infiltrated controls. Figure S3. Col‐0 plants infiltrated with TRV‐GUS, TRV‐FIGL1 and TRV‐RECQ4 do not display significant differences in the number of viable or aborted seeds per silique in comparison to non‐infiltrated Col‐0 controls. Figure S4. Model fit of negative binomial GLMM for Col‐0 msh4 shows differences in the predicted value for the variable ‘viable seeds per silique’ in infiltrated plants with TRV‐RECQ4 and TRV‐FIGL1 as compared to controls. Figure S5. Phenotypic comparison of Ler msh4 plants infiltrated with TRV‐RECQ4 and TRV‐FIGL1 reveals an increase in silique length and seed numbers as compared to Ler msh4 controls and Ler::TRV‐GUS. Figure S6. Model fit of negative binomial GLMM for Ler msh4 shows differences in the predicted value for the variable ‘viable seeds per silique’ in infiltrated plants with TRV‐RECQ4 and TRV‐FIGL1 as compared to controls. Figure S7. Pair‐plot shows a direct correlation between the variable ‘number of tetrads’ per day of sampling and per individual with the variables ‘number of positive flowers’ and ‘total number of flowers’ in Col‐0 plants treated with TRV‐QRT2 and monitored for 13 consecutive days. Figure S8. Photobleaching in Ler::TRV‐PDS plants and absence of TRV1 and TRV2 expression in A. thaliana offspring obtained from treated plants. [file TPJ-111-19-s003.zip › tpj15733-sup-0001-FigureS1.pdf]

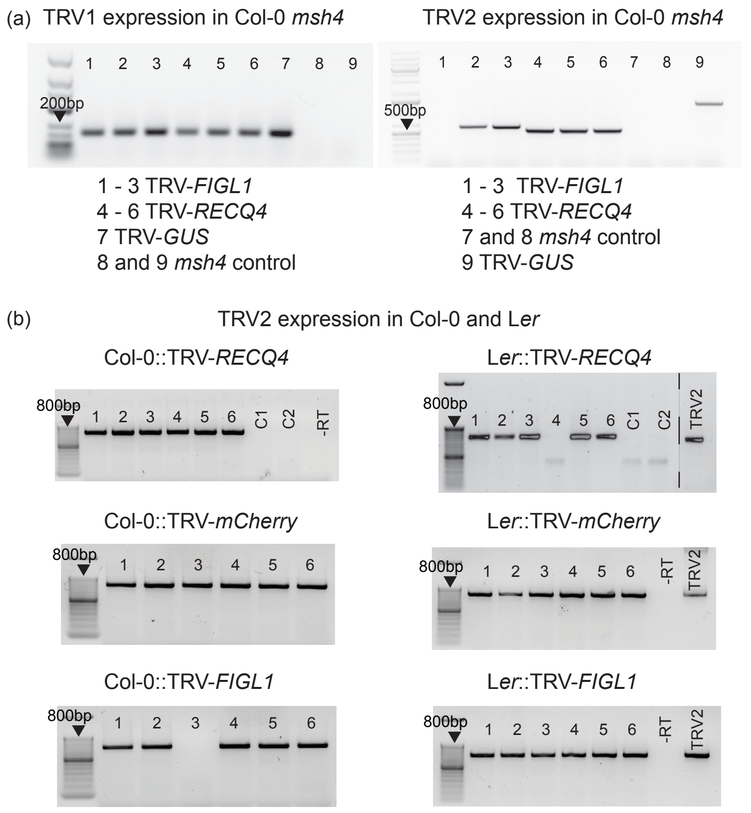
(a) TRV1 expression in Col-0 msh4
TRV2 expression in Col-0 msh4
1 2 3 4 5 6 7 8 9
200bp
500bp
1 - 3 TRV-FIGL1
4 - 6 TRV-RECQ4
7 TRV-GUS
8 and 9 msh4 control
1 - 3 TRV-FIGL1
4 - 6 TRV-RECQ4
7 and 8 msh4 control
9 TRV-GUS
(b) TRV2 expression in Col-0 and Ler
Col-0::TRV-RECQ4
800bp
1 2 3 4 5 6 C1 C2 -RT
Ler::TRV-RECQ4
800bp
1 2 3 4 5 6 C1 C2 TRV2
Col-0::TRV-mCherry
800bp
1 2 3 4 5 6
Ler::TRV-mCherry
800bp
1 2 3 4 5 6 -RT TRV2
Col-0::TRV-FIGL1
800bp
1 2 3 4 5 6
Ler::TRV-FIGL1
800bp
1 2 3 4 5 6 -RT TRV2
