# Supplementary material for: Knock‐down of gene expression throughout meiosis and pollen formation by virus‐induced gene silencing in Arabidopsis thaliana
Source: Plant J. 2022 Jun 18;111(1):19–37. doi: 10.1111/tpj.15733 (PMC9543169; doi:10.1111/tpj.15733)
Supplement: Supplementary file 1 — Figure S1. sqRT‐PCR analyses in infiltrated and non‐infiltrated plants to detect TRV1 and TRV2 expression in flower buds of Col‐0 and Ler plants infiltrated with TRV‐FIGL1, TRV‐RECQ4, TRV‐GUS and TRV‐mCherry but not in untreated controls. Figure S2. Col‐0 plants infiltrated with TRV‐RECQ4, TRV‐FIGL1 and TRV‐GUS do not display shortened siliques or differences in the number of well‐developed seeds as compared to Col‐0 non‐infiltrated controls. Figure S3. Col‐0 plants infiltrated with TRV‐GUS, TRV‐FIGL1 and TRV‐RECQ4 do not display significant differences in the number of viable or aborted seeds per silique in comparison to non‐infiltrated Col‐0 controls. Figure S4. Model fit of negative binomial GLMM for Col‐0 msh4 shows differences in the predicted value for the variable ‘viable seeds per silique’ in infiltrated plants with TRV‐RECQ4 and TRV‐FIGL1 as compared to controls. Figure S5. Phenotypic comparison of Ler msh4 plants infiltrated with TRV‐RECQ4 and TRV‐FIGL1 reveals an increase in silique length and seed numbers as compared to Ler msh4 controls and Ler::TRV‐GUS. Figure S6. Model fit of negative binomial GLMM for Ler msh4 shows differences in the predicted value for the variable ‘viable seeds per silique’ in infiltrated plants with TRV‐RECQ4 and TRV‐FIGL1 as compared to controls. Figure S7. Pair‐plot shows a direct correlation between the variable ‘number of tetrads’ per day of sampling and per individual with the variables ‘number of positive flowers’ and ‘total number of flowers’ in Col‐0 plants treated with TRV‐QRT2 and monitored for 13 consecutive days. Figure S8. Photobleaching in Ler::TRV‐PDS plants and absence of TRV1 and TRV2 expression in A. thaliana offspring obtained from treated plants. [file TPJ-111-19-s003.zip › tpj15733-sup-0010-Supinfo.pdf]

Supporting information

Figure S1. **sqRT-PCR analyses of TRV1 and TRV2 in inoculated and non-inoculated plants. (a)** Shows TRV1 and TRV2 expression in *msh4* Col-0 plants inoculated with TRV-*FIGL1*, TRV-*RECQ4,* the control construct TRV-*GUS* and control Col-0 *msh4*. Note that while TRV1 and TRV2 are always detected in all the inoculated plants with the exception of TRV2 in *msh4*::TRV-*FIGL1* #Plant 1, neither of them are detected in non-inoculated controls. **(b)** Depicts TRV2 expression in Col-0 and L*er* plants inoculated with TRV-*mCherry*, TRV-*FIGL1* and TRV-*RECQ4* while TRV2 is never detected in non-inoculated control plants (C1 and C2). As a positive control for TRV-*mCherry*, TRV-*FIGL1*, or TRV-*RECQ4* we used as template the original TRV2 construct corresponding to each target.

Figure S2. Col-0 plants inoculated with TRV-*RECQ4*, TRV-*FIGL1* and TRV-*GUS* do not display signs of sterility. (a) Shows a comparison for silique elongation along the main branch between a non-inoculated Col-0 plant and those treated with TRV-*FIGL1,* TRV-*RECQ4* and TRV-*GUS*. Scale bar 2 cm. **(b)** Depicts siliques selected from main and lateral branches of Col-0 control and Col-0 treated plants. No evident size variation is observed between the different siliques of each group. Scale bar 1 cm. **(c)** Shows a comparison regarding seed development and seed numbers found in siliques of control Col-0 and Col-0 treated plants. No significant reduction in the seed set was observed in these plants (**Table S1**). Scale bar 0.2 cm.

Figure S3. The Box-plots represent the number of viable and aborted seeds per silique found in Col-0 wild-type plants and Col-0 plants infiltrated with TRV-*RECQ4*, TRV-*FIGL1,* TRV-*GUS*. Each box-plot represents a single plant and each point, a single silique for Col-0 (n=60), Col-0::TRV-*RECQ4* (n=60), Col-0::TRV-*FIGL* (n=60) , Col-0::TRV-*GUS* (n=40). Note that the outliers are indicated with a red dot. Since our statistical analysis methods are sensitive to outliers, these data points are excluded from further data analysis as per the protocol described in Zuur *et al.,* (2010).

Figure S4. Visual representation of the negative binomial GLMM [viable seeds per silique ~ treatment + accession + (1| plant ID)] for Col-0 *msh4* inoculated and non-inoculated plants. R^2 of the model is 0.678. Each plot represents a single plant and each plot shows in black dots the observed data points (seeds per silique), and in red dot the values predicted by the model. This visual plot shows how well the model is fitted to the data by catering a different prediction value for each plant. More specifically, the position of red diamonds differs for each plant due to the model adjusting the intercept for each plant in order to fit the observations more closely. Although each plant can only be given a treatment, there are four red diamonds per plot as the regression produces a prediction for every level of the treatment category: *msh4* (n=13) and *msh4*::TRV-*GUS* controls (n=4), *msh4*::TRV-*FIGL1* (n=10) and msh4::TRV-*RECQ4* (n=10).

Figure S5. The L*er* *msh4* Inoculated plants with TRV-*FIGL1* and TRV-*RECQ4* show rescue of the semi-sterile phenotype. (a) The control plants *msh4* and *msh4*::TRV-*GUS* display short siliques along the main stem length, whereas *msh4* plants inoculated with TRV-*FIGL1* and TRV-*RECQ4* produce long siliques. Scale bar 2 cm. **(b)** Displays a close-up comparison of siliques from the same plant of *msh4* and *msh4*::TRV-*GUS* controls with short siliques versus the siliques obtained from *msh4*::TRV-*FIGL1* and a *msh4*::TRV-*RECQ4* plants, which show size variation and longer siliques. Scale bar 1 cm. **(c)** Siliques selected from a *msh4*::TRV-*FIGL1* and *msh4*::TRV-*RECQ4* show a clear rescue of the semi-sterile phenotype with a larger seed-set in comparison with *msh4* and *msh4*::TRV-*GUS*. Scale bar 0,2 cm.

Figure S6. Model fit of negative binomial GLMM [viable seeds per silique ~ treatment + accession + (1| plant ID)] for L*er msh4* inoculated and non-inoculated plants. R^2=0.678. Each plot represents a single plant and each plot shows in black dots the observed data points (seeds per silique), and in red dot the values predicted by the model. This visual plot shows how well the model is fitted to the data by catering a different prediction value for each plant. More specifically, the position of red diamonds differs for each plant due to the model adjusting the intercept for each plant in order to fit the observations more closely. Although each plant can only be given a treatment, there are four red diamonds per plot as the regression produces a prediction for every level of the treatment category: *msh4* (n=8) and *msh4*::TRV-*GUS* controls (n=4), *msh4*::TRV-*FIGL1* (n=8) and *msh4*::TRV2-*RECQ4* (n=8).

Figure S7. Phenotyping of plants treated with TRV-*QRT2* reveals a possible correlation between the total 'number of tetrads' per day of sampling and per individual plant with five other variables. The variable 'Plant ID' refers to each individual plant from 1 to 16 considered during the sampling time. The variable 'Day' refers to the specific day of sampling, from 1 to 13,. 'Positive n. of flowers' and 'Total n. of flowers' represent in both cases the total number of flowers for each category per plant and day of sampling. The variable 'First positive day' shows the first day in which each plant produced pollen tetrads.

Figure S8: L*er*::TRV-*PDS* plants produce transgene-free offspring. (a) Ler plants infiltrated with TRV-*PDS* show photobleaching in several tissues including siliques, which are indicated on the figure with white arrows. **(b)** Neither TRV1 nor TRV2-derived transcripts could be detected by sqRT-PCR in offspring obtained from white siliques of L*er*::TRV-*PDS* (n=30). Samples obtained from inflorescences of Ler::TRV-*mCherry* serve as positive control for the detection of TRV1 and TRV2.
