# Supplementary material for: Knock‐down of gene expression throughout meiosis and pollen formation by virus‐induced gene silencing in Arabidopsis thaliana
Source: Plant J. 2022 Jun 18;111(1):19–37. doi: 10.1111/tpj.15733 (PMC9543169; doi:10.1111/tpj.15733)
Supplement: Supplementary file 2 — Table S1. Model summary for binomial (logit) GLMM: odds of viable over aborted seeds per silique ~ treatment + (1| plant ID) for non‐infiltrated Col‐0 and infiltrated Col‐0 with TRV‐RECQ4, TRV‐FIGL1 and TRV‐GUS. Table S2. Model summary for negative binomial GLMM: viable seeds per silique ~ treatment + (1| plant ID) for Col‐0 and Ler msh4 mutants. Table S3. Model summary for negative binomial GLMM for normalized tetrad counts from QRT2 knock‐down: normalized tetrads ~ day + (1| plant ID). Table S4. List of primers used to create and confirm by Sanger sequencing the generated VIGS constructs and assess TRV1 and TRV2 expression by semi‐quantitative real‐time PCR (sqRT‐PCR) and digital PCR (dPCR). [file TPJ-111-19-s002.pdf]

Supporting information

Table S1. It shows the odds of obtaining viable seeds over aborted seeds in Col-0 plants treated with the three TRV constructs after analyzing the data shown in Data S1. Model summary for binomial (logit) GLMM equation is: odds of viable over aborted seeds per silique ~ treatment + (1| plant ID). Nagelkerke's Pseudo-R^2 (Cragg and Uhler) is 0.0173. Low R^2 value indicates that the TRV treatment only explains 1.7% of the variance in seed counts. The intercept corresponds to Col-0 untreated controls.

	Estimate	Standard error	z-value	p
**Intercept**	4.217290	0.196615	21.449	< 0.001***
**TRV-*GUS***	0.001604	0.219729	0.007	0.994
**TRV-*FIGL1***	-0.120962	0.191093	-0.633	0.527
**TRV-*RECQ4***	0.253337	0.209516	1.209	0.227

Significant codes: *** for 0.001, ** for 0.01, * for 0.05

Table S2. It shows the model counts of viable seeds in Col-0 and L*er msh4* plants treated with the three TRV constructs. Treatment with TRV-*FIGL1* and TRV-*RECQ4* is predicted to yield 2.875 and 3.68 more seeds per siliques than *msh4* while holding 'accession' constant, respectively. If the background is L*er* instead of Col-0, 2.749 more seeds per silique are expected. Model summary for negative binomial GLMM: viable seeds per silique ~ treatment + accession + (1| plant ID) for Col-0 and L*er msh4* mutants. R^2 is 0.678. The estimates and standard error presented here are exponentiated from model estimates to obtain actual seed counts. The intercept corresponds to Col-0 *msh4.*

	Estimate	Standard error	z-value	p
**Intercept**	3.126768	1.120416	10.031	< 0.001***
**TRV-*GUS***	0.8101791	1.219694	-1.060	0.289
**TRV-*FIGL1***	2.875136	1.156386	7.270	< 0.001***
**TRV-*RECQ4***	3.684740	1.154884	9.059	< 0.001***
**L*er msh4***	2.748898	1.114382	9.337	< 0.001***

Significant codes: *** for 0.001, ** for 0.01, * for 0.05

Table S3. Model summary for negative binomial GLMM for normalized tetrad counts obtained after a 13-day phenotyping period of 16 plants treated with TRV-*QRT2*. The model equation is the following: normalized tetrads ~ day + (1| plant ID). R^2 is 0.098. The link function is logarithmic. The intercept represents the first day of sampling.

	Estimate	Standard error	Z-value	p
**Conditional model**				
**Intercept**	1.64009	0.39481	4.154	< 0.001***
**Day**	0.19742	0.03839	5.142	< 0.001***
**Zero-inflation model**				
**Intercept**	-1.2494	0.4077	-3.064	< 0.01**

Significant codes: *** for 0.001, ** for 0.01, * for 0.05

Table S4. Primer list used to generate and confirm by Sanger sequencing of the TRV constructs and assess TRV1, TRV2, *FIGL1*, *RECQ4A* and *RECQ4B* expression by semi-quantitative real-time PCR (sqRT-PCR) and digital PCR (dPCR). For the cloning of TRV constructs, restriction recognition sites are introduced in each fragment and added

to the corresponding primer sequence. The enzymes that recognize these sites are indicated in the primer name. The orientation of the fragment cloned into TRV2 is indicated as (s) for sense or (as) for antisense.

**Purpose**	**Primer name**	**Sequence**
TRV-*mCherry* cloning (as)	*mCherry*_Fw_XbaI	CATCTAGAACATCCTGTCCCCTCAGTTC
	*mCherry*_Rw_EcoRI	CAGAATTCGGTTTTCTTCTGCATTACGG
TRV-FIGL1 cloning (as)	*FIGL1*_Fw_BamHI	CAGGATCCACCACCGGAGTTTCTGAATG
	*FIGL1*_Rw_XbaI	CATCTAGATAGAAGACGAAGCCCAAGGA
TRV-RECQ4 cloning (s)	*RECQ4*_Fw_XhoI	CACTCGAGTGGTATTCTGAAGCAGAAATT
	*RECQ4*_Rw_SmaI	CACCCGGGCAATATATAATTCCACACTCA
TRV-*QRT2* cloning (as)	*QRT2*_Fw_SmaI	CACCCGGGGCGGTACAAGTGAGCAATGT
	*QRT2*_Rw_XhoI	CACTCGAGAACATTTCCTCGGGTGTCCA
TRV-*OSD1* cloning (as)	*OSD1*_Fw_XbaI	CATCTAGAGGAGCTGGGACTGGAGGAGAC
	*OSD1*_Rw_EcoRI	CAGAATTCTGAGTAGCTTCTTCTCGGGTG
TRV2 sequencing	TRV2_seq	GATGGACATTGTTACTCAAGGAAG
TRV1 sqRT-PCR	RT_TRV1_Fw	CATGTTGGTGGGAAGAAGAGTGAACACAAG
	RT_TRV1_Rw	GATTTGAATGAACCCAGGCGTATCTGCAG
	TRV1_Fw	CTTGAAGAAGAAGACTTTCGAAGTCTC
	TRV1_Rw	GTAAAATCATTGATAACAACACAGACAAAC
TRV2 sqRT-PCR	TRV2_Fw	GGTCAAGGTACGTAGTAGAG
	TRV2_Rw	CGAGAATGTCAATCTCGTAGG
AT3G47060 sqRT - dPCR	*AT3G47060*_Fw	GGCTTGGTGCTCAACTTGAAGAG
	*AT3G47060*_Rw	TGGTGCAACCACCATGCTTAAC
At4G26410 sqRT - dPCR	*At4G26410*_Fw	GAGCTGAAGTGGCTTCCATGAC
	*At4G26410*_Rw	GGTCCGACATACCCATGATCC
PDS5 sqRT-PCR	*PDS5A*_Fw	ATGGCTCAGAAGCCGGAGGAACAGTTGAAAG
	*PDS5A*_Rw	CTTAACCAACGTCTTGATCCCATATATCTTC
TRV-*mCherry* dPCR	*mCherry*_Fw_dPCR	CCGACATCCCCGACTACTTG
	*mCherry*_Rw_dPCR	TGTAGATGAACTCGCCGTCC
FIGL1 dPCR	*FIGL1*_dPCR_Fw	TGGCCATTGTTGCGTCCTGA
	*FIGL1*_dPCR_Rw	CACCTTCGCCAATCCACTTGC
RECQ4A dPCR	*RECQ4A*_dPCR_Fw	TACCCCACAGCCATCAGTGT
	*RECQ4A*_dPCR_Rw	TCTGGACAAAGCCCCAACTTT
RECQ4B dPCR	*RECQ4B*_dPCR_Fw	CGACCTGACTACCAGGGTCTT
	*RECQ4B*_dPCR_Rw	CAGTTTACCAGCCCGAGAGC
